# Engineering Multi‐Site Platinum Ensembles Synergistically Boosts Catalysis

**DOI:** 10.1002/advs.202415937

**Published:** 2025-02-18

**Authors:** Tao Dong, Fei Xiao, Xuanning Wu, Tao Ban, Jian Ji, Biyuan Liu, Jiarui Zhang, Jiuxing Jiang, Dieqing Zhang, Weixing Yang, Gaoyuan Liu, Xin Yang, Haibao Huang

**Affiliations:** ^1^ School of Environmental Science and Engineering Sun Yat‐sen University Guangzhou 510006 China; ^2^ College of Ecology and Environment School of Chemical Engineering and Technology Xinjiang University Urumqi 830017 China; ^3^ School of Resources and Environment Nanchang University Nanchang 330031 China; ^4^ MOE Key Laboratory of Bioinorganic and Synthetic Chemistry School of Chemistry Sun Yat‐sen University Guangzhou 510006 China; ^5^ The Education Ministry Key Lab of Resource Chemistry College of Chemistry and Materials Science Shanghai Normal University Shanghai 200234 China; ^6^ Midea Group Foshan 528300 China

**Keywords:** heterogeneous catalysis, multi‐type active sites, Pt ensembles, reactive oxygen species

## Abstract

Engineering stable and efficient noble metal ensembles with multi‐type active sites while understanding the role of each site at the atomic level remains a significant challenge in heterogeneous catalysis. Herein, a sub‐nanometric Pt ensemble catalyst with a diverse array of active sites is constructed via a dual‐confinement strategy, which exhibits superior activity and durability with minimal Pt loading (0.13 wt.%). Simultaneously, the roles of different Pt sites at the atomic scale are determined through in situ characterization methods and density functional theory (DFT) calculations. Specifically, Pt top sites predominantly serve as pivotal centers for O═O bond activation, whereas Pt−O−Si interfacial sites primarily govern the activation of H─OH and C─H bonds. The reactive oxygen species (O_2_^−^, O_2_^2−^, and −OH) generated from O_2_ and H_2_O activation synergistically enhance formaldehyde (HCHO) oxidation and shorten the reaction pathway. This study sheds light on a better understanding and rational design of catalyst active sites via precise synthesis of multi‐site ensembles or discerning the distinct contributions of various catalytic sites.

## Graphical abstract

Pt ensemble catalysts with abundant types and quantities of active sites are designed. The Pt top sites and Pt−O−Si interfacial sites synergistically accelerate the adsorption/activation of O_2_ and H_2_O, generating multi‐active oxygen species (O_2_^−^, O_2_^2−^, and ─OH), and ultimately achieve ≈100% removal of formaldehyde (HCHO) at room temperature.

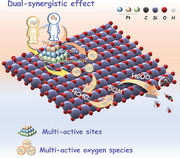

## 1 Introduction

Synergistic collaboration among researchers and institutions in large‐scale scientific projects, such as the Human Genome Project (HGP) and the development of the Large Hadron Collider (LHC), demonstrates the critical role of synergistic interactions in driving significant progress and success in human society. Similarly, in the microscopic particle world of heterogeneous catalysis, the synergistic interactions among catalytic sites, which aim to reduce costs and enhance efficiency, are also crucial. Particularly in complex catalytic reactions, chemical bond cleavage, and formation are frequently dominated by specific metal active sites, which require synergistic interactions between distinct catalytic sites to obtain high catalytic activity and selectivity.^[^ ^1^, ^2^ ^]^ This synergistic interaction among catalytic sites, known as the “ensemble effect” was described by Sachtler et al. in the 1970s.^[^ ^3^ ^]^ For example, formaldehyde (HCHO), a highly toxic indoor pollutant, has garnered significant attention from the International Agency for Research on Cancer (IRAC) due to its potential to cause severe health hazards, highlighting the urgent need for effective removal.^[^ ^4^, ^5^ ^]^ Catalytic oxidation of HCHO is a common yet crucial area involving the cleavage of various chemical bonds, including O═O, H─OH, and C─H bonds.^[^ ^6^ ^]^ It is evident that a single type of active site is generally insufficient for the simultaneous activation of all bonds due to competitive adsorption among the reactant molecules.^[^ ^7^ ^]^ Consequently, designing catalysts with multiple functional ensemble sites is significantly important for maintaining high catalytic performance in complex reactions.

In recent years, a substantial amount of research has been devoted to the design of catalyst active sites, such as single atoms and nanoclusters.^[^ ^8^, ^9^, ^10^ ^]^ This is particularly for supported noble metal catalysts, emerging as a pivotal research focus in the areas of hydrogen production,^[^ ^11^ ^]^ carbon monoxide (CO) oxidation,^[^ ^12^ ^]^ organic synthesis,^[^ ^13^ ^]^ and volatile organic compounds (VOCs) degradation.^[^ ^14^ ^]^ For instance, Xie et al.^[^ ^15^ ^]^ prepared a Pd‐Cu dual sites cluster catalyst that exhibits excellent C_2+_ selectivity in CO_2_ reduction reaction. Zhang et al.^[^ ^1^ ^]^ prepared a supported Rh catalyst with both isolated atoms and ensemble sites that exhibits excellent catalytic efficiency in the conversion of cyclohexanol to phenol. However, a significant challenge remains in designing noble metal ensembles at the atomic scale to expose multi‐type active sites while ensuring their stability. Currently, modulating the interactions between noble metals and support to achieve the construction and stability of active sites is the most frequently studied and effective strategy.^[^ ^16^, ^17^, ^18^ ^]^ Typically, local interactions between noble metal and support materials are established through a high‐temperature reduction or oxidation treatment to stabilize active sites.^[^ ^19^, ^20^ ^]^ Nevertheless, when noble metals are dispersed at the scale of single atoms and nanoclusters, they often exhibit poor temperature resistance due to the increased surface free energy.^[^ ^21^ ^]^ This makes single atoms or nanoclusters readily agglomerate into large particles even in the existence of strong metal‐support interactions or even at room temperature, resulting in a decrease in active sites and subsequent catalyst deactivation.^[^ ^22^, ^23^ ^]^ Therefore, maintaining abundant types and quantities of active sites to achieve an ensemble effect is key for the long‐term efficient operation of catalysts.

Based on this, researchers are committed to developing more effective methods to stabilize and disperse noble metal ensembles onto specific anchoring sites of the support materials. So far, 3D flaky cross‐linked structured oxides or hierarchical porous materials (e.g., TiO_2_, Al_2_O_3_, SiO_2_, zeolite) have emerged as promising candidates for stabilizing noble metal active sites due to their unique geometric structures, physical characteristics, and tunable surface sites.^[^ ^24^, ^25^ ^]^ In particular, the 3D nanoflower mesoporous SiO_2_ (NFM‐SiO_2_) has attracted extensive attention due to its abundant mesoporous structure and surface hydroxyl groups (─OH), unique sheet cross‐linked structure, and high thermal stability.^[^ ^26^ ^]^ The lamellar surfaces are rich in anchoring sites—surface hydroxyl groups—that can robustly bind to noble metals and form new types of active sites, such as metal‐support interfacial sites (e.g., metal–O–Si or metal–Si sites).^[^ ^6^, ^27^, ^28^ ^]^ In addition, the spatial confinement provided by the SiO_2_ layers acts as an effective barrier, substantially blocking the migration of noble metals and maintaining the abundance of active sites.^[^ ^26^ ^]^ From this perspective, NFM‐SiO_2_ would exhibit outstanding advantages in the construction of multi‐type noble metal active sites while simultaneously maintaining the stability of the noble metal ensembles.

In this study, we precisely designed and synthesized a sub‐nanometric Pt ensemble catalyst with multi‐type active sites stable on a 3D NFM‐SiO_2_ surface (named Pt@NFM‐SiO_2_‐400) via an interface and surface space dual‐confinement strategy. The Pt@NFM‐SiO_2_‐400 exhibited excellent catalytic activity and durability of HCHO oxidation with minimal Pt loading (0.13 wt.%), which can be attributed to the synergistic interactions among the various active sites. Aberration‐corrected scanning transmission electron microscopy (AC‐STEM) revealed that the structural configuration and aggregation morphology of Pt ensembles undergo dynamic migration and redistribution at varying reduction temperatures. Surprisingly, uniformly dispersed sub‐nanometric Pt clusters were exclusively obtained at a reduction temperature of 400 °C. Furthermore, the extended X‐ray absorption fine structure (EXAFS), CO‐adsorption diffuse reflectance infrared Fourier transform spectroscopy (CO‐DRIFTS), and DFT calculations confirmed the Pt ensembles with multi‐type active sites, such as Pt top sites, Pt side sites, and Pt−O−Si interfacial sites. Specifically, the Pt top sites are the activation site of O_2_, while the Pt−O−Si interfacial sites serve as the primary activation for HCHO and H_2_O. Mechanistic studies demonstrated that the active species O_2_^−^, O_2_^2−^, and ─OH, generated from the activation of O_2_ and H_2_O, synergistically promote the effective catalytic oxidation of HCHO, achieving 100% degradation at room temperature and shortening the reaction pathway. This work inspires innovative approaches for the rational design of efficient heterogeneous catalysts.

## 2 Results and Discussion

### 2.1 Construction, Characterization, and Dynamic Transformation of Pt Ensembles

**Figure 1:**
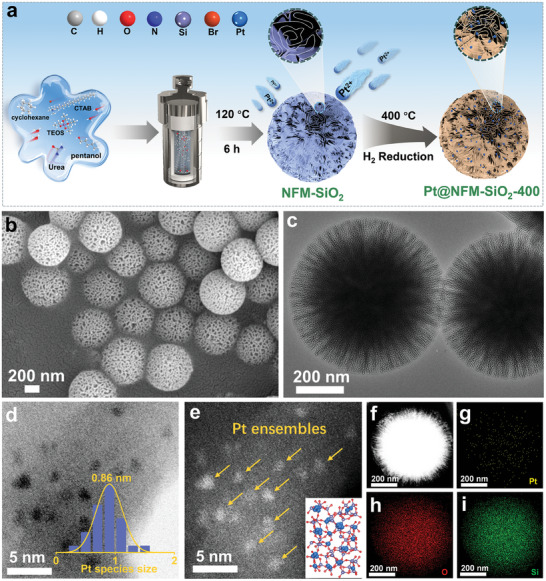
a) Schematic illustration for the synthesis, b) SEM, c) TEM, d) AC‐TEM, Pt size distribution (inset in figure d), e) AC‐STEM, structure schematic (inset in figure e), f) HAADF‐STEM, and g–i) EDS‐mapping images of Pt@NFM‐SiO_2_‐400.

We constructed Pt ensemble catalysts via a facile wetness impregnation method, using abundant surface ─OH sites and the spatial barrier effect between NFM‐SiO_2_ layers to dual‐confine Pt species (marked as Pt@NFM‐SiO_2_‐400), as illustrated in **Figure** **1**a. Scanning electron microscopy (SEM) and transmission electron microscopy (TEM) images (Figures S1 and S2, Supporting Information) reveal that NFM‐SiO_2_ exhibits a 3D lamellar cross‐linked nanoflower structure. After loading Pt species and reducing treatment, the morphology of NFM‐SiO_2_ remained unchanged (Figure 1b,c). Aberration‐corrected scanning transmission electron microscopy (AC‐STEM) shows that the Pt species are primarily dispersed as sub‐nanometric clusters on SiO_2_ layers (Figure 1d,e). The mean size of Pt ensembles is measured at 0.86 nm, with a corresponding structural schematic shown in Figure 1e. For comparison, closed mesoporous silica spheres (MCM‐41) without lamellar cross‐linked structure and conventional silica spheres (SiO_2_) without mesoporous structure were also prepared and employed as supports for loading Pt species. SEM images show that MCM‐41 and SiO_2_ are spherical in shape (Figures S3 and S4, Supporting Information). The Pt species are dispersed on both MCM‐41 and SiO_2_ as Pt nanoparticles, with mean sizes of 2.42 and 2.53 nm, respectively (Figures S5 and S6, Supporting Information). This indicates that the 3D NFM‐SiO_2_ is beneficial for anchoring and dispersing Pt species into sub‐nanoscale aggregates, thus exposing more active sites. Elemental energy‐dispersive X‐ray spectroscopy (EDS) mapping images confirm a homogeneous distribution of Pt species on the surfaces of 3D NFM‐SiO_2_, MCM‐41, and SiO_2_ (Figure 1f–i; Figures S7 and S8, Supporting Information). The inductively coupled plasma optical emission spectrometry (ICP‐OES) analysis confirmed that the Pt content of three samples is as low as ≈0.13 wt.% (Table S1, Supporting Information).

X‐ray diffraction (XRD) patterns (Figure S9, Supporting Information) show that all silicon spheres are composed of amorphous silica. Additionally, the characteristic crystal plane diffraction peaks corresponding to Pt (111) and (200) are evident in Pt/SiO_2_,^[^ ^29^ ^]^ but not detectable in Pt@NFM‐SiO_2_‐400 and Pt/MCM‐41‐400. This observation demonstrates that mesoporous silicas (NFM‐SiO_2_ and MCM‐41) facilitate the dispersion of Pt species. N_2_ physical adsorption displays that mean mesopore sizes for NFM‐SiO_2_ and MCM‐41 are 3.4 and 2.1 nm (Figure S10 and Table S1, Supporting Information), respectively. Fourier transform infrared (FT‐IR) spectroscopy spectra (Figure S11, Supporting Information) display a series of characteristic peaks of typical silica materials, such as isolated SiOH* (3744 cm^−1^),^[^ ^30^ ^]^ Si─OH─Si (3648 cm^−1^),^[^ ^31^ ^]^ Si─OH (1627 and 932 cm^−1^),^[^ ^30^, ^32^, ^33^ ^]^ and Si─O─Si (1220, 1042, and 808 cm^−1^).^[^ ^34^, ^35^, ^36^ ^]^ The abundant silicon ─OH groups are more favorable for anchoring and dispersing Pt species.

To understand the effects of temperature on the dynamic structural transformation of Pt ensembles and to determine the optimal temperature for forming Pt sub‐nanometric clusters, we investigated the surface microstructure changes of Pt@NFM‐SiO_2_ from 200 to 800 °C using AC‐STEM. As shown in Figure S12 (Supporting Information), at a reduction temperature of 200 °C, some Pt clusters and a few Pt nanoparticles are distributed in some areas over NFM‐SiO_2_ layers, with a mean size of 1.25 nm. By increasing the reduction temperature to 300 °C, a substantial number of Pt nanoparticles and clusters appear on the whole silica sphere (Figure S13, Supporting Information), with the mean size of Pt species increasing to 2.13 nm. When the reduction temperature increases to 400 °C, the Pt species are redispersed into uniform sub‐nanometric clusters with a mean size of 0.86 nm and reach the minimum value (Figure 1d,e). Nevertheless, at a further elevated reduction temperature of 500 °C, the Pt clusters aggregated into larger nanoparticles, although a large number of small Pt clusters remained (Figure S14, Supporting Information), resulting in a mean size of 3.44 nm for the Pt species.

Interestingly, as the reduction temperature increases to 600 °C, larger Pt particles begin to redisperse into smaller Pt particles, and the number of Pt clusters is decreased, with the mean size of Pt species dropping to 3.29 nm (Figure S15, Supporting Information). Upon raising the reduction temperature to 700 °C, Pt species further dispersed into smaller uniform nanoparticles with a mean size of 2.74 nm (Figure S16, Supporting Information), while only a few Pt clusters existed. Conversely, at 800 °C, Pt clusters disappeared and reaggregated into larger nanoparticles, with a mean size of 3.01 nm (Figure S17, Supporting Information). These findings suggest that the size of Pt ensembles is temperature‐sensitive and undergoes a dynamic transformation as the temperatures rise, as illustrated in Figure S18 (Supporting Information). Notably, at temperatures below 700 °C, Pt species coexist as both nanoclusters and nanoparticles, whereas at temperatures exceeding 700 °C, the nanoclusters disappear and Pt nanoparticles dominate. Surprisingly, the formation of homogeneous sub‐nanometric Pt clusters only occurs at an optimal temperature of 400 °C, thereby maintaining or exposing more active sites.

### 2.2 Determining the Active Site Types, Coordination, and Valence State of Pt Ensembles

**Figure 2:**
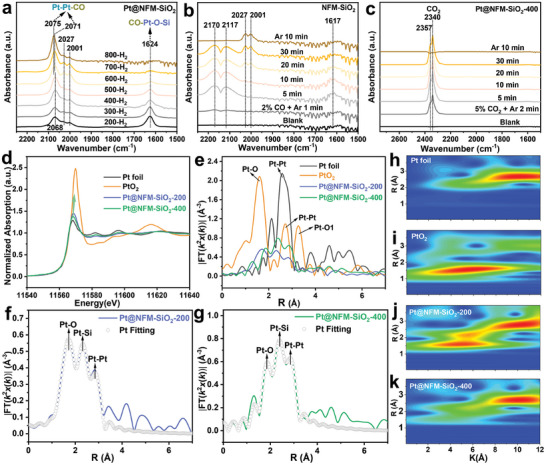
In situ DRIFTS of CO adsorption on a) Pt@NFM‐SiO_2_ (under different reduction temperatures) and b) NFM‐SiO_2_. c) In situ DRIFTS of CO_2_ adsorption on Pt@NFM‐SiO_2_‐400. The comparison of Pt L3‐edge d) XANES and e) EXAFS spectra. Pt L3‐edge EXAFS spectra of f) Pt@NFM‐SiO_2_‐200 and g) Pt@NFM‐SiO_2_‐400. The wavelet transforms from experimental data for h) Pt foil, i) PtO_2_, j) Pt@NFM‐SiO_2_‐200, and k) Pt@NFM‐SiO_2_‐400.

To further investigate the active site types, coordination, valence state, and dynamic structural transformation of Pt ensembles at the atomic level, we combined in situ CO‐DRIFTS, X‐ray photoelectron spectroscopy (XPS), X‐ray absorption fine structure (EXAFS), and DFT calculations to clarify Pt site types and corresponding role in activation chemical bonds. As shown in **Figure** **2**a, the bands at 2068–2075 cm^−1^ can be attributed to the linear adsorption peak of CO on metallic Pt species.^[^ ^37^, ^38^ ^]^ A progressive increase in the adsorption intensity of this peak was noted, accompanied by a shift to higher wavenumbers with the rising temperature, indicative of the aggregation of Pt species. It is reported that the peaks at 2027 and 2001 cm^−1^ can be assigned to the vibration modes of gaseous CO adsorbed within mesoporous channels,^[^ ^39^ ^]^ and the peaks at 1624 cm^−1^ can be attributed to CO adsorption on M─O─Si interfacial sites where M represents metal atoms.^[^ ^40^ ^]^ To validate these observations, a CO‐DRIFTS adsorption experiment was conducted over NFM‐SiO_2_, with detailed methodologies outlined in the Supporting Information. As can be seen from Figure 2b, four adsorption peaks appeared after adsorption of 2% CO/Ar for 30 min, while only two adsorption peaks of 2027 and 2001 cm^−1^ remained after purging by Ar gas for 10 min. This is because the peaks at 2170 and 2117 cm^−1^ are ascribed to gas‐phase CO molecules,^[^ ^41^ ^]^ while the peaks at 2026 and 2001 cm^−1^ are attributed to strong adsorption of CO within mesoporous channels. However, the absence of the CO adsorption peak near 1624 cm^−1^ prior to Pt species loading implies CO adsorption on a specific Pt site.

Previous studies have also shown that CO_3_^2−^ is easily adsorbed on the catalyst surface, often resulting in peaks near 1624 cm^−1^.^[^ ^42^ ^]^ To exclude this factor, in situ CO_2_‐DRIFTS was measured over Pt@NFM‐SiO_2_‐400 to determine the presence of CO_3_^2−^ species on the sample surface. As shown in Figure 2c, only gaseous CO_2_ adsorption peaks (2357 and 2340 cm^−1^) are detected,^[^ ^43^ ^]^ with no peaks at 1624 cm^−1^, effectively excluding the influence of CO_3_^2−^ species. These findings suggest that the peak at 1624 cm^−1^ can be attributed to the vibrational mode of CO adsorption on Pt─O─Si interfacial sites (CO─Pt─O─Si). Furthermore, the intensity of this peak diminishes as the reduction temperature increases, ultimately disappearing at 800 °C. This phenomenon likely results from the cleavage of the Pt─O─Si bond, coupled with the migration and aggregation of Pt clusters into larger nanoparticles as temperature rises, aligning with the AC‐STEM results under different reduction temperatures.

XPS spectra were measured to investigate the effects of reduction temperature on the state and valence transformation of Pt ensembles. As shown in Figure S19a (Supporting Information), it can be observed that only the Pt^2+^ species (75.11 and 72.10 eV) can be detected over PtO_x_@NFM‐SiO_2_.^[^ ^19^, ^44^ ^]^ After a reduction treatment (10% H_2_/Ar) at 200 °C, the Pt 4f_7/2_ peak for Pt@NFM‐SiO_2_‐200 can be deconvoluted into two peaks at ≈72.20 and 71.28 eV, corresponding to Pt^2+^ and Pt^0^ species, respectively.^[^ ^19^, ^44^ ^]^ Meanwhile, the ratio of Pt^0^/(Pt^0^ + Pt^2+^) gradually increases as the temperature rises from 200 to 500 °C (Figure S19b–e, Supporting Information), suggesting a reduction of the Pt^2+^ species to the Pt^0^, while some PtO_x_ species remain unreduced. Subsequently, Pt^2+^ species are predominantly reduced to Pt^0^ after reaching a reduction temperature of 600 °C or higher (700 and 800 °C; Figure S19f–h, Supporting Information).

To gain insight into the elemental coordination, electronic state, and bonding structure of Pt ensembles on the surface of Pt@NFM‐SiO_2_ catalysts, X‐ray absorption near‐edge structure (XANES) and extended X‐ray absorption fine structure (EXAFS) tests were performed on Pt@NFM‐SiO_2_‐200 and Pt@NFM‐SiO_2_‐400 samples. As shown in Figure 2d, the overall trend of the Pt L3‐edge XANES spectra of Pt@NFM‐SiO_2_‐200 and Pt@NFM‐SiO_2_‐400 samples is similar to that of metal Pt foil, suggesting the existence of Pt^0^ species. Furthermore, the white line peak (11570 eV) can be used to determine the change of valence state in Pt species, with higher valence states corresponding to increased peak intensity.^[^ ^45^ ^]^ The peak intensity of Pt@NFM‐SiO_2_‐200 exceeds that of Pt@NFM‐SiO_2_‐400, suggesting that the Pt ensembles in Pt@NFM‐SiO_2_‐200 exhibit a higher valence state than Pt@NFM‐SiO_2_‐400, and some PtO_x_ species remained unreduced, consistent with the XPS results. The Pt L3‐edge EXAFS spectra (Figure 2e–g), reveal the existence of Pt─O, Pt─Si, and Pt─Pt bonds in both samples. Combining the fitting results (Table S2, Supporting Information), it is obtained that the Pt─Pt bond coordination numbers of Pt@NFM‐SiO_2_‐200 (3.7 ± 0.1) and Pt@NFM‐SiO_2_‐400 (4.8 ± 0.3) are smaller than the Pt foil (12) and PtO_2_ (9.2 ± 0.9), implying a reduction in Pt particle size and the presence of Pt clusters, as also supported by AC‐STEM results. In addition, the existence of Pt─O and Pt─Si bonds in both samples implies a robust interaction between Pt and SiO_2_, potentially leading to the formation of Pt─O─Si interfacial sites. Wavelet transform analysis (Figure 2h–k) shows a decrease in signal intensities for Pt─O and Pt─Si bonds, alongside an increase in Pt─Pt bonds as reduction temperature rises from 200 to 400 °C, signifying a higher degree of reduction for Pt@NFM‐SiO_2_‐400 compared to Pt@NFM‐SiO_2_‐200.

**Figure 3:**
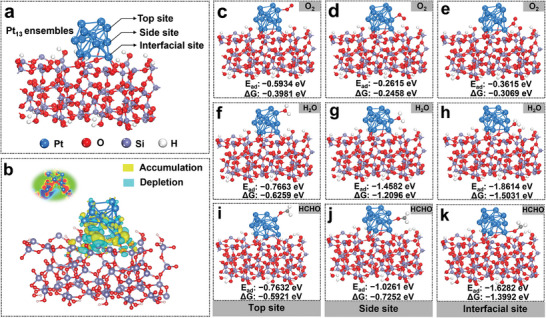
a) Pt_13_ ensembles‐SiO_2_ optimal model and b) corresponding charge density distributions. The c–e) O_2_, f–h) H_2_O, and i–k) HCHO molecules adsorbed on different sites, and corresponding adsorption energies and Gibbs free energies.

Collectively, the AC‐STEM, CO‐DRIFTS, and EXAFS results elucidate that the sub‐nanometric Pt@NFM‐SiO_2_‐400 ensemble catalyst has multi‐type active sites, such as Pt─Pt metal sites and Pt─O─Si interfacial sites. In the context of formaldehyde oxidation, the reactants, including O_2_, H_2_O, and HCHO, and the adsorption/activation mechanism of each reactant at different Pt sites remains unclear. It is significant to study the roles of different active sites and reactants in the design of high‐performance noble metal Pt catalysts. Therefore, the DFT calculations were performed to investigate the electronic structure of catalysts and the adsorption behavior of O_2_, H_2_O, and HCHO molecules at different Pt sites. A model comprising 13 Pt atoms was constructed (**Figure** **3**a), categorizing the Pt─O─Si site bonded to SiO_2_ as the “Interfacial site,” the adjacent Pt site as the “Side site,” and the topmost metallic Pt site as the “Top site.”. DFT analysis (Figure 3b) reveals substantial electron transfer between Pt and SiO_2_, with electrons migrating from the metallic Pt site to the Pt─O─Si interfacial site. It implies that Pt and O atoms have strong bonding cooperation, and the Pt─O─Si interfacial site can function as an efficient electron transport channel.

Furthermore, the adsorption characteristics of O_2_, H_2_O, and HCHO molecules at each active site (Figure 3c–k and Figure S20, Supporting Information) are studied. It shows that O_2_ exhibits the highest adsorption energy (*E*_ad_ = −0.5934 eV) at the top site, with the lowest Gipps free energy of adsorption (Δ*G* = −0.3981 eV), indicating a preference for O_2_ adsorption at this site. Furthermore, H_2_O and HCHO demonstrate preferential adsorption at the Pt─O─Si interfacial site, with H_2_O showing the highest adsorption energy (*E*_ad_ = −1.8614 eV) and lowest Gipps free energy (Δ*G* = −1.5031 eV), followed closely by HCHO (*E*_ad_ = −1.6282 eV, Δ*G* = −1.3992 eV). These findings indicate the Pt top site mainly serves as the center for O_2_ adsorption/activation, while the Pt─O─Si interfacial site is crucial for the H_2_O and HCHO adsorption/activation. Based on the above results, we successfully constructed a Pt ensemble catalyst with multi‐type active sites, which can synergistically accelerate the activation of various chemical bonds in the HCHO oxidation reaction.

### 2.3 Catalytic Performance and High Activity Origins in Pt Ensemble Sites

**Figure 4:**
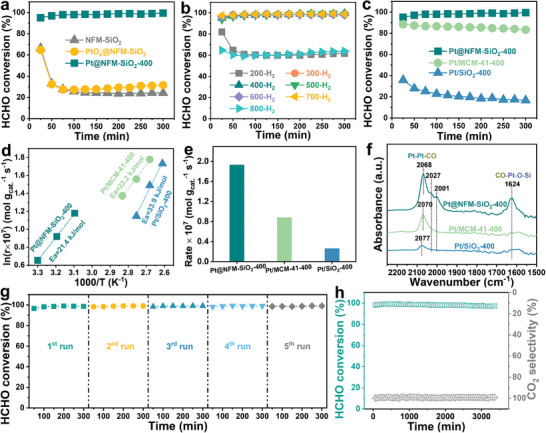
a) HCHO oxidation over NFM‐SiO_2_, PtO_x_@NFM‐SiO_2_, and Pt@NFM‐SiO_2_‐400. b) HCHO oxidation over Pt@NFM‐SiO_2_ under different reduction temperatures. c) HCHO oxidation, d) Arrhenius plots, e) rate, and f) CO‐DRIFTS over Pt@NFM‐SiO_2_‐400, Pt/MCM‐41‐400, and Pt/SiO_2_‐400. g) Cycling and h) stability test of Pt@NFM‐SiO_2_‐400.

To demonstrate the unique advantages and high activity origins of Pt ensemble catalysts in HCHO oxidation, a series of catalytic performance tests and characterizations were performed. The activity of catalysts for oxidation HCHO was evaluated in an air‐flow containing 100 ppm HCHO with weight hourly space velocity (WHSV) and relative humidity (RH) of 120 000 mL g_cat._^−1^ h^−1^ and 40%, respectively. As shown in **Figures** **4**a and S21 (Supporting Information), the HCHO conversion over NF‐SiO_2_ is 24%, with no CO_2_ selectivity, indicating that NF‐SiO_2_ only adsorbs HCHO. After loading Pt species, the HCHO conversion and CO_2_ selectivity are gradually improved over the PtO_x_@NFM‐SiO_2_ sample (calcinated under a 20% O_2_/N_2_ atmosphere at 400 °C), but it cannot deeply oxidize HCHO. However, the HCHO oxidation activity of Pt@NFM‐SiO_2_‐400 is significantly enhanced after a 10% H_2_/Ar reduction at 400 °C. It shows that the HCHO conversion and CO_2_ selectivity over Pt@NFM‐SiO_2_‐400 are both increased to ≈100%, indicating metallic Pt^0^ species have a higher HCHO removal effect than oxidized PtO_x_ species. The HCHO oxidation performance of Pt@NFM‐SiO_2_ samples at different reduction temperatures was further studied, and the test results are shown in Figure 4b. It is observed that, compared with the PtO_x_@NFM‐SiO_2_ sample, the HCHO conversion increased to ≈61% after 200 °C reduction. Furthermore, when the reduction temperature increased to 300 °C, the HCHO conversion greatly increased to ≈100%, and the activity remained stable until 700 °C. However, the HCHO conversion dramatically decreased to ≈64% after the temperature rose to 800 °C. Unexpectedly, the CO_2_ selectivity is maintained at 100% and remained unchanged with the reduction temperature (Figure S22, Supporting Information). The results suggest that the reduced Pt@NFM‐SiO_2_ samples can deeply and efficiently oxidize the captured HCHO into CO_2_ and H_2_O, preventing the accumulation of intermediates on the catalyst surface.

To elucidate the influence of reduction temperature on catalyst activity, a series of characterization analyses were carried out to investigate the bonding structure, crystal structure, and pore structure of Pt@NFM‐SiO_2_ at different reduction temperatures. FT‐IR analysis (Figure S23, Supporting Information) indicates a decline in the peak intensities of Si─OH─Si and Si─OH, alongside an increase in isolated SiOH^*^, suggesting a reduction in surface ─OH groups that facilitate the dispersion and anchoring of Pt species, causing the Pt species aggregate and overall types and quantities of active sites to be reduced. This is consistent with the above AC‐STEM and CO‐DRIFTS results of Pt@NFM‐SiO_2_ at different temperatures. In addition, the XRD pattern (Figure S24, Supporting Information) reveals a gradual weakening of the amorphous SiO_2_ peak intensity, indicative of a reduction in Si─OH and Si─OH─Si bonds. Nevertheless, no diffraction peaks corresponding to Pt species are detected, although the temperature rose to 800 °C, indicating that the Pt species would aggregate but might still be highly dispersed. It can be observed that the specific surface area gradually decreases from 470 to 304 m^2^ g^−1^ as the temperature rises from 200 to 800 °C, but the loading of Pt species (≈0.14 wt.%) is almost unchanged (Figure S25 and Table S1, Supporting Information).

Combining with the AC‐STEM, CO‐DRIFTS, FT‐IR, XRD, BET, and ICP analyses, it can be concluded that the reduction temperature range of 200–800 °C unchanged the Pt content but significantly affected the types and quantities of active sites. Although aggregation of Pt species occurs in the range of 300–700 °C, sufficient active sites remain available for efficient deep oxidation of O_2_, H_2_O, and HCHO. However, insufficient reduction at low temperatures over Pt@NFM‐SiO_2_‐200 limits the availability of metallic Pt sites for O_2_ activation, while excessive temperatures lead to the loss of Pt─O─Si interfacial sites and reduce activation sites for H_2_O and HCHO, ultimately lowering overall catalytic efficiency.

To highlight the unique advantages of the Pt species supported by NFM‐SiO_2_ mesoporous materials, the HCHO degradation properties of Pt@NFM‐SiO_2_‐400, Pt/MCM‐41‐400, and Pt/SiO_2_‐400 samples were further compared, and the test results are shown in Figure 4c and Figure S26 (Supporting Information). The Pt@NFM‐SiO_2_‐400 ensemble catalyst exhibited optimal HCHO degradation performance, achieving ≈100% conversion and CO_2_ selectivity, while Pt/MCM‐41‐400 and Pt/SiO_2_‐400 demonstrated lower conversion of ≈90%, and declining activity and selectivity, indicative of lower stability. Kinetic tests (Figure 4d,e and Table S3, Supporting Information) were performed to further study the catalytic performance of related catalysts, and the internal and external diffusion have been eliminated. It can be found that the Pt@NFM‐SiO_2_‐400 ensemble catalyst has the lowest activation energy (21.4 kJ mol^−1^) and the highest reaction rate (r) and turnover frequency (TOF) values compared with Pt/MCM‐41‐400 and Pt/SiO_2_‐400. This indicates that the Pt@NFM‐SiO_2_‐400 ensemble catalyst exhibits optimal HCHO degradation capacity.

In addition, the HCHO oxidation performance of Pt/MCM‐41 and Pt/SiO_2_ after 700 °C high‐temperature reduction (marked as Pt/MCM‐41‐700 and Pt/SiO_2_‐700) was also tested. It can be observed that the HCHO oxidation performance of Pt/MCM‐41‐700 and Pt/SiO_2_‐700 significantly decreased (Figure S27, Supporting Information). The XRD pattern in Figure S28 (Supporting Information) shows that the obvious diffraction peaks of Pt (111) and Pt (200) crystal planes can be detected on Pt/MCM‐41‐700 and Pt/SiO_2_‐700, indicating that Pt/MCM‐41 and Pt/SiO_2_ have poor sintering resistance. In contrast, Pt@NFM‐SiO_2_ maintains ≈100% HCHO elimination even after high‐temperature (700 °C) reduction. Based on the above results, it can be proven that NFM‐SiO_2_ mesoporous material outperforms MCM‐41 and SiO_2_ in dispersing and anchoring Pt ensemble sites. Therefore, Pt@NFM‐SiO_2_‐400 has better HCHO degradation performance and sintering resistance. Furthermore, we compared the catalytic performance for HCHO oxidation of various oxide‐supported Pt catalysts with literature. As shown in Table S4 (Supporting Information), Pt@NFM‐SiO_2_ exhibits excellent catalytic activity and stability for HCHO oxidation over the Pt‐based catalysts.

To further identify the primary reasons why Pt@NFM‐SiO_2_‐400 ensemble catalyst has optimal catalytic performance compared to Pt/MCM‐41‐400 and Pt/SiO_2_‐400, the Pt species state, redox ability, and oxygen/water activation ability were studied. As shown in Figure S29 (Supporting Information), all samples showed two valence Pt species (Pt^0^ and Pt^2+^). The ratio of Pt^0^/(Pt^0^ + Pt^2+^) in Pt@NFM‐SiO_2_‐400 remained unchanged after the HCHO oxidation test (marked as Pt@NFM‐SiO_2_‐400‐used). Nevertheless, the ratio of Pt^0^/(Pt^0^ + Pt^2+^) over Pt/MCM‐41‐400 and Pt/SiO_2_‐400 declined from 0.74 and 0.61 to 0.65 and 0.52, respectively, after the HCHO oxidation test (marked as Pt/MCM‐41‐400‐used and Pt/SiO_2_‐400‐used). These results indicate that Pt@NFM‐SiO_2_‐400 has excellent oxidation resistance at low temperatures owing to the dual confinement effect of NFM‐SiO_2_. The CO‐DRIFTS spectra (Figure 4f) show that two types of Pt sites can be observed at Pt@NFM‐SiO_2_‐400: metallic Pt site (2068 cm^−1^) and Pt─O─Si interfacial site (1624 cm^−1^). In contrast, only metallic Pt sites (2070 and 2077 cm^−1^) were detected in Pt/MCM‐41‐400 and Pt/SiO_2_‐400, and the peak intensity is weaker than Pt@NFM‐SiO_2_‐400. This result suggests that the Pt@NFM‐SiO_2_‐400 ensemble catalyst can expose more types of active sites to oxidation HCHO.

In the H_2_‐TPR test, as shown in Figure S30 (Supporting Information), the positive peaks at 40–103 °C are attributed to the reduction of Pt^2+^ to Pt^0^,^[^ ^46^ ^]^ and the negative peaks at 86–133 °C are associated with the decomposition of PtH_x_.^[^ ^47^ ^]^ It can be clearly observed that the Pt^2+^ reduction peak and PtH_x_ decomposition peak of Pt@NFM‐SiO_2_‐400 are the lowest, implying that it has optimal reducibility. The peaks at 480–504 °C corresponded to the reduction of bulk PtO_x_ to metallic Pt,^[^ ^47^ ^]^ and the peak intensity is minimum over Pt@NFM‐SiO_2_‐400. The results revealed that there are barely large Pt particles on the Pt@NFM‐SiO_2_‐400 surface, existing instead as tiny nanoclusters, which aligns with the AC‐STEM results (Figure 1d,e). As can be seen in the O_2_‐TPO test (Figure S31, Supporting Information), the negative peaks at 94–111 °C can be attributed to the desorption peak of chemisorbed oxygen.^[^ ^48^, ^49^ ^]^ Among the catalysts, Pt@NFM‐SiO_2_‐400 has the lowest desorption temperature, indicating its enhanced O_2_ activation capability. In addition, the peaks at 230 and 488 °C are attributed to the oxidation of surface and bulk metallic Pt species, respectively.^[^ ^50^ ^]^ The peak at 625 °C is assigned to the re‐decomposition of PtO_x_ species into metallic Pt and O atoms. The results of H_2_‐TPR and O_2_‐TPO show that Pt@NFM‐SiO_2_‐400 has better redox ability.

To gain a deeper insight into the catalyst activation gaseous O_2_ and H_2_O capacity and the dynamic changes of reactive oxygen species on catalyst surfaces, we carried out the EPR, O_2_‐DRIFTS, and H_2_O‐DRIFTS studies. As shown in Figure S32 (Supporting Information), the superoxide (O_2_^−^) species could be detected at a g value of 2.016.^[^ ^51^ ^]^ O_2_‐DRIFTS (Figure S33, Supporting Information) that the bonds at 974, 980, and 984 cm^−1^ could be attributed to O_2_^−^ species,^[^ ^52^ ^]^ consistent with the EPR results. Furthermore, the peroxide species (O_2_^2−^) were observed at 885, 886, and 907 cm^−1^.^[^ ^52^ ^]^ The relative intensities (Figure S33d, Supporting Information) of surface reactive oxygen species (O_2_^−^ and O_2_^2−^) on Pt@NFM‐SiO_2_‐400 reached their highest levels after 30 min of O_2_ adsorption, indicating Pt@NFM‐SiO_2_‐400 has the better O_2_ activation capacity. In the H_2_O‐DRIFTS (Figure S34, Supporting Information), absorption peaks at 3662–3673 cm^−1^ can be attributed to surface ─OH vibrations, while the vibration peaks at 3216–3268 cm^−1^ can be attributed to the absorbed H_2_O.^[^ ^53^ ^]^ As can be seen from Figure S34d (Supporting Information), it proved that Pt@NFM‐SiO_2_‐400 has the better H_2_O activation capacity due to the highest levels of ─OH species after 30 min of H_2_O adsorption. Based on the above characterization results, the Pt@NFM‐SiO_2_‐400 ensemble catalyst exhibits the best HCHO catalytic oxidation performance, which can be attributed to the satisfactory oxidation resistance and more Pt site types and quantities, as well as the better redox capacity. This accelerates the O_2_ and H_2_O adsorption/activation to form abundant reactive oxygen species (O_2_^−^, O_2_^2−^, and ─OH), which achieved the efficient and deep degradation of HCHO.

Additionally, the service life of the Pt@NFM‐SiO_2_‐400 ensemble catalyst was measured at different test conditions to evaluate its potential application value. As shown in Figure S35a (Supporting Information), at a relative humidity (RH) is 40%, the HCHO conversion reaches ≈100%. Subsequently, a slight decrease in conversion was observed when RH dropped to 20%, and a significant decline occurred at 10% RH. This decline might be due to insufficient surface ─OH species for the HCHO oxidation under low RH conditions, causing a decrease in catalyst activity. Amazingly, the HCHO conversion recovered to 100% after the RH rose back to 40%, indicating that Pt@NFM‐SiO_2_‐400 has excellent regeneration. However, the HCHO conversion almost remained unchanged under high humidity RHs of 60% and 80%, demonstrating Pt@NFM‐SiO_2_‐400 has excellent moisture resistance and can efficiently adsorb and activate H_2_O. It can be observed that the CO_2_ selectivity (Figure S35b, Supporting Information) is maintained at ≈100% under different humidity conditions, illustrating HCHO can be deeply oxidized to CO_2_ and H_2_O over the Pt@NFM‐SiO_2_‐400 surface. As shown in Figure S36 (Supporting Information), the catalytic oxidation performance of HCHO over Pt@NFM‐SiO_2_‐400 remained unchanged at various concentrations. It implies that the Pt@NFM‐SiO_2_‐400 catalyst has broad adaptability to HCHO concentrations. Furthermore, the HCHO conversion (Figure S37a, Supporting Information) decreased after the WHSV was increased to 240000 and 360000 mL g_cat._^−1^ h^−1^, owing to the short residence time of the reactants at high WHSV and insufficient contact time with the active sites. However, the CO_2_ selectivity (Figure S37b, Supporting Information) still remained ≈100%, indicating the Pt@NFM‐SiO_2_‐400 catalyst could deeply oxidize the captured HCHO molecules. As shown in Figure S38 (Supporting Information), the HCHO conversion and CO_2_ selectivity maintained ≈100% with the temperature change (25–80 °C). This result indicates that Pt@NFM‐SiO_2_‐400 has excellent adaptability to ambient temperatures. The cyclic and long‐term stability tests were also performed to study the durability of Pt@NFM‐SiO_2_‐400. As shown in Figure 4g and Figure S39 (Supporting Information), the HCHO conversion and CO_2_ selectivity still maintain ≈100% after five cyclic tests, indicating that Pt@NFM‐SiO_2_‐400 has excellent durability. After a 3360‐min test, the HCHO removal efficiency remains at ≈98% (Figure 4h), further confirming excellent durability and potential application value for the Pt@NFM‐SiO_2_‐400 ensemble catalyst.

### 2.4 Reaction Mechanism Studies

**Figure 5:**
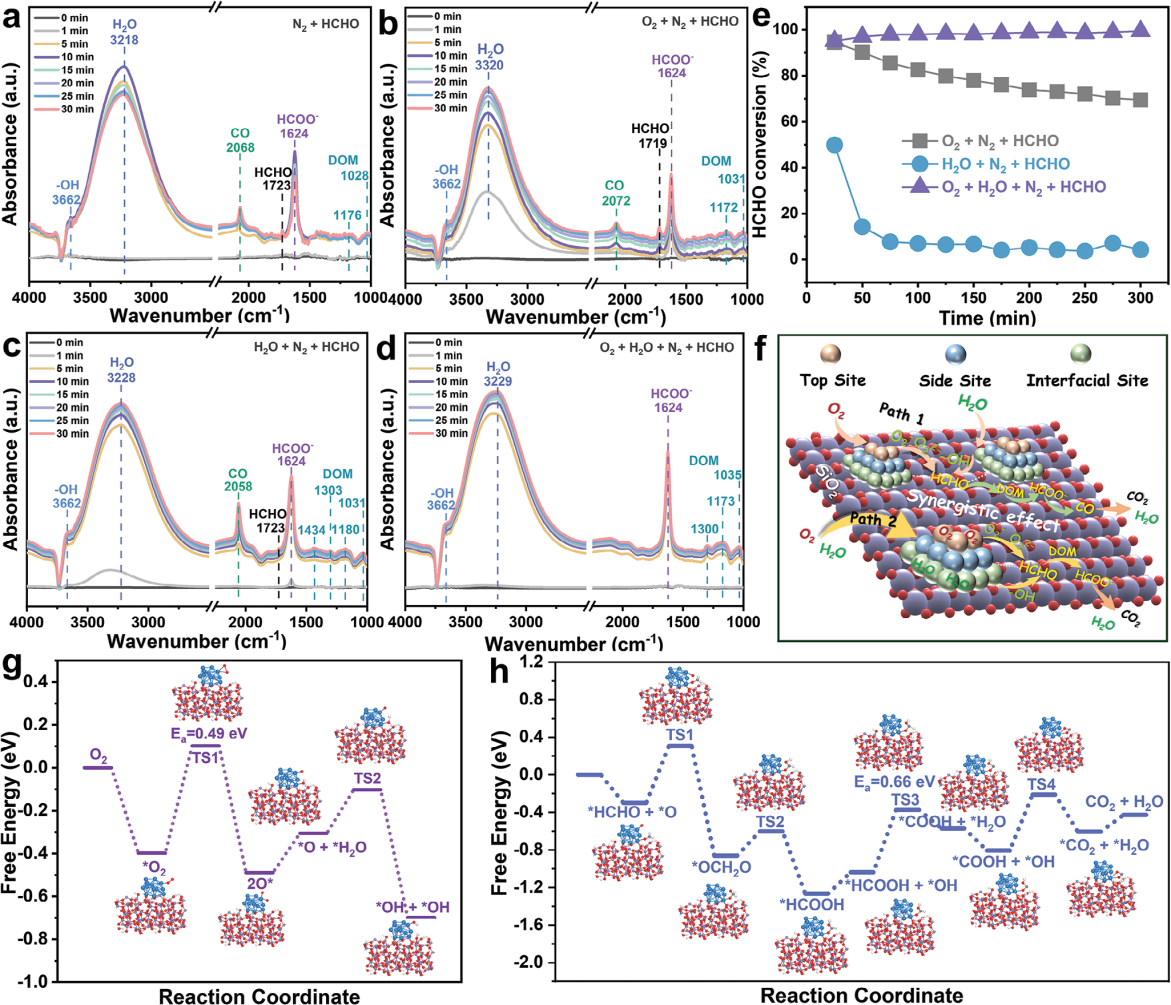
In situ DRIFTS spectra of HCHO oxidation over Pt@NFM‐SiO_2_‐400 under different reaction atmospheres: a) N_2_ + HCHO, b) O_2_ + N_2_ + HCHO, c) H_2_O + N_2_ + HCHO, and d) O_2_ + H_2_O + N_2_ + HCHO. e) HCHO conversion over Pt@NFM‐SiO_2_‐400 under different reaction atmospheres. f) Possible reaction pathways of HCHO oxidation over Pt@NFM‐SiO_2_‐400. Gibbs free energy calculations for g) O_2_ and H_2_O active, and h) HCHO oxidation.

To determine the HCHO oxidation reaction pathways over Pt@NFM‐SiO_2_‐400, in situ DRIFTS spectra were used to study the reaction intermediates. The detailed testing procedures are presented in the Supporting Information, and the assigned results are present in Table S5 (Supporting Information). As shown in **Figure** **5**a, after exposure to N_2_ + HCHO (100 ppm) for 30 min, the absorption vibration peak of HCHO (1723 cm^−1^) and some typical intermediate species vibration peaks can be detected.^[^ ^54^ ^]^ Such as surface ─OH (3662 cm^−1^),^[^ ^53^ ^]^ H_2_O (3218 cm^−1^),^[^ ^53^ ^]^ CO (2068 cm^−1^),^[^ ^37^, ^38^ ^]^ DOM (1176 and 1028 cm^−1^),^[^ ^6^, ^53^, ^55^, ^56^ ^]^ and HCOO^−^ (1624 cm^−1^) species.^[^ ^6^, ^53^, ^55^, ^56^ ^]^ It is evident that the peak intensities of CO, DOM, and HCOO^−^ species gradually increased, but the peak intensities of H_2_O and ─OH first increased and then decreased. This indicates that the surface ─OH of the catalyst is involved in HCHO oxidation in the absence of O_2_ and H_2_O. And the H_2_O from the product will be re‐activated into ─OH to participate in the reaction, causing peak intensities to increase and then weaken.

After the atmosphere is switched to O_2_ + N_2_ + HCHO, as shown in Figure 5b, the intermediate species types of ─OH (3662 cm^−1^), CO (2072 cm^−1^), DOM (1172 and 1031 cm^−1^), and HCOO^−^ (1624 cm^−1^) remained unchanged. However, the peak intensities of CO are weaker than those in Figure 5a, indicating that gaseous O_2_ plays an indispensable role in HCHO oxidation. Since O_2_ can provide additional reactive oxygen species (O_2_^−^ and O_2_^2−^) to accelerate the degradation of intermediate species. Furthermore, after the atmosphere is switched to H_2_O + N_2_ + HCHO (RH = 40%; Figure 5c), an increase in the number of DOM peaks (1434, 1303, 1180, and 1031 cm^−1^) and a stronger CO peak (2058 cm^−1^) were observed, indicating that the ─OH species activated by physical adsorption water participated in the HCHO oxidation, and H_2_O also plays a vital role. Surprisingly, the peaks of the intermediate species CO disappear after the reaction atmosphere is switched to O_2_ + H_2_O + N_2_ + HCHO (RH = 40%), as shown in Figure 5d, and only the ─OH (3662 cm^−1^), DOM (1300, 1173, and 1035 cm^−1^) and HCOO^−^ (1624 cm^−1^) can be observed. It is demonstrated that the simultaneous presence of O_2_ and H_2_O can change the HCHO oxidation reaction pathway.

Based on the above results, in the HCHO reaction system, either O_2_ or H_2_O alone is unfavorable to the HCHO oxidative degradation and follows a CO‐containing path: HCHO → DOM → HCOO^−^ → CO → CO_2_. Only when the simultaneous existence of O_2_ and H_2_O can provide sufficient reactive oxygen species (O_2_^−^, O_2_^2−^, and ─OH) for the HCHO oxidation. Among them, O_2_^−^ and O_2_^2−^ species mainly activate the initial HCHO into DOM, and then ─OH oxidizes the DOM into HCOO^−^, finally oxidized to CO_2_ and H_2_O. The reactive oxygen species synergistically promote HCHO oxidation, thus shortening the HCHO oxidation pathway and improving catalytic efficiency. Furthermore, the HCHO activity tests under different atmospheres (Figure 5e) show that Pt@NFM‐SiO_2_‐400 has poor HCHO oxidation activity under O_2_ or H_2_O alone. It is clearly found that Pt@NFM‐SiO_2_‐400 has excellent HCHO oxidation performance under the simultaneous existence of O_2_ and H_2_O. This result further proved that the reactive oxygen species O_2_^−^, O_2_^2−^, and ─OH produced by O_2_ and H_2_O synergistically promoted the efficient deep oxidation of HCHO. The possible reaction pathways of HCHO oxidation over Pt@NFM‐SiO_2_‐400 are shown in Figure 5f.

To further clarify the O_2_, H_2_O, and HCHO oxidation reaction mechanisms, as well as the evolution process of intermediate species over the Pt@NFM‐SiO_2_‐400 surface. DFT calculations were performed to study the reaction pathways and free energies of O_2_, H_2_O, and HCHO activation. As shown in Figure 5g, O_2_ is preferentially adsorbed at the top site to form O_2_^*^ species. Subsequently, these O_2_^*^ species are activated into two O^*^ species, and then one O^*^ species reacts with H_2_O^*^ adsorbed at the Pt─O─Si interfacial site to form two OH^*^ species. Among them, TS1 (*E*_a_ = 0.49 eV) has the highest energy barrier, which is the rate‐determining step of the O_2_ and H_2_O activations. Furthermore, the reaction pathway involving reactive oxygen species (O^*^ and OH^*^) in the synergistic oxidation of HCHO was calculated, as shown in Figure 5h. First, O^*^ species reacts with HCHO^*^ adsorbed at the Pt─O─Si interface site to form CH_2_O_2_^*^ species, which is further transformed into HCOOH^*^ species. Then, HCOOH^*^ reacts with OH^*^ to produce COOH^*^ species. Finally, COOH^*^ reacts with OH^*^ to produce CO_2_ and H_2_O. Among the four transition states, TS3 (*E*_a_ = 0.66 eV) presents the highest energy barrier. This finding indicates that HCOOH^*^ reacting with OH^*^ to form COOH^*^ is the rate‐determining step of HCHO oxidation. Therefore, experimental results and DFT calculations clearly demonstrate a dual‐synergistic effect exists in HCHO oxidation over the Pt@NFM‐SiO_2_‐400 surface. Initially, Pt top sites and Pt─O─Si interfacial sites can synergistically promote the activation of O_2_ and H_2_O to generate abundant reactive oxygen species (O_2_^−^, O_2_^2−^, and ─OH), and then O_2_^−^, O_2_^2−^, and ─OH species further synergistically enhance the degradation of key intermediate species (DOM and HCOO^−^) to CO_2_ and H_2_O.

## 3 Conclusion

In summary, we successfully synthesized sub‐nanometric Pt ensembles with multi‐type active sites through a dual‐confinement strategy, where these sites can synergistically accelerate reactant activation. The size of Pt ensembles undergoing dynamic transformation between clusters and nanoparticles at different reduction temperatures. The smallest sub‐nanometer Pt clusters are obtained at a reduction temperature of 400 °C, which has optimal redox ability, catalytic activity, durability, and adaptability to various working conditions in HCHO oxidation. In addition, CO‐DRIFTS and EXAFS demonstrated that the Pt ensembles have multi‐type active sites, including Pt─Pt metal sites and Pt─O─Si interface sites. DFT calculation results prove that different Pt sites play different roles in the activation of chemical bonds. Particularly, the Pt top site is the O_2_ activation center, while the Pt─O─Si interfacial site is the HCHO and H_2_O activation centers. The mechanism study showed that the generated O_2_^−^, O_2_^2−^, and ─OH from the activation of O_2_ and H_2_O synergistically facilitated the effective catalytic oxidation of HCHO and shortened the reaction pathway. This study not only established the controllable preparation method of high‐performance Pt ensemble catalysts but also helped us understand the roles of different metal active sites in heterogeneous catalysts.

## Conflict of Interest

The authors declare no conflict of interest.

## Supporting information



Supporting Information

## Data Availability

The data that support the findings of this study are available in the supplementary material of this article.
