# Supplementary material for: Engineering Multi‐Site Platinum Ensembles Synergistically Boosts Catalysis
Source: Adv Sci (Weinh). 2025 Feb 18;12(14):2415937. doi: 10.1002/advs.202415937 (PMC11984847; doi:10.1002/advs.202415937)
Supplement: Supplementary file 1 — Supporting Information [file ADVS-12-2415937-s001.pdf]

ADVANCED SCIENCE
Open Access

Supporting Information

for *Adv. Sci.*, DOI 10.1002/advs.202415937

Engineering Multi-Site Platinum Ensembles Synergistically Boosts Catalysis

*Tao Dong, Fei Xiao, Xuanning Wu, Tao Ban, Jian Ji, Biyuan Liu, Jiarui Zhang, Jiuxing Jiang, Dieqing Zhang, Weixing Yang, Gaoyuan Liu, Xin Yang and Haibao Huang**

Supporting Information

Engineering multi-site platinum ensembles synergistically boosts catalysis

Tao Dong[1], Fei Xiao[1], Xuanning Wu[1], Tao Ban[2], Jian Ji[3], Biyuan Liu[1], Jiarui Zhang[1], Jiuxing Jiang[4], Dieqing Zhang[5], Weixing Yang[6], Gaoyuan Liu[6], Xin Yang[1], and Haibao Huang[1,2]*

[1]School of Environmental Science and Engineering, Sun Yat-sen University, Guangzhou 510006, China

[2]College of Ecology and Environment, School of Chemical Engineering and Technology, Xinjiang University, Urumqi 830017, China

[3]School of Resources and Environment, Nanchang University, Nanchang, 330031, China

[4]MOE Key Laboratory of Bioinorganic and Synthetic Chemistry, School of Chemistry, Sun Yat-Sen University, Guangzhou 510006, China

[5]The Education Ministry Key Lab of Resource Chemistry, College of Chemistry and Materials Science, Shanghai Normal University, Shanghai 200234, China

[6]Midea Group, Foshan 528300, China

*E-mail: seabao8@gmail.com

List of Contents

Experimental Procedures 1
Catalyst preparation 1
Catalyst characterization 2
Activity test 3
Reaction kinetics measurement 4
Density functional theory calculations 5
In situ DRIFTS measuring processes 5
CO-DRIFTS adsorption 5
O_2-DRIFTS adsorption 6
H_2O-DRIFTS adsorption 6
HCHO in situ DRIFTS adsorption and oxidation at 25 °C 6
Supplementary Tables 7
Table S1. Physicochemical properties and Pt content of catalysts measured by N_2 sorption isotherms and ICP. 7
Table S2. EXAFS fitting parameters at the Pt L3-edge for various samples (S_0^2=0.86) 8
Table S3. Kinetic catalytic performances of Pt@NFM-SiO_2-400 and related catalysts.[a] 9
Table S4. Comparison with literatures about the performances of Pt based catalysts for HCHO oxidation. 10
Table S5. Summary of the vibrational modes and positions of surface species in the oxy-carbon surface species region 11
Supplementary Figures 12
Figure S1. SEM images of NFM-SiO_2. 12
Figure S2. TEM images of NFM-SiO_2. 13
Figure S3. SEM images of Pt/MCM-41-400. 14
Figure S4. SEM images of Pt/SiO_2-400. 15
Figure S5. TEM images and Pt NPs size distribution of Pt/MCM-41-400. 16
Figure S6. TEM images and Pt NPs size distribution of Pt/SiO_2-400. 17
Figure S7. HAADF-STEM and EDS-Mapping of Pt/MCM-41-400 18
Figure S8. EDS-Mapping of Pt/SiO_2-400 19
Figure S9. XRD patterns of Pt@NFM-SiO_2-400 and related catalysts. 20
Figure S10. N_2 adsorption/desorption isotherms of Pt@NFM-SiO_2-400 and related catalysts 21
Figure S11. FT-IR spectra of Pt@NFM-SiO_2-400 and related catalysts 22
Figure S12. AC-TEM images and Pt NPs size distribution of Pt@NFM-SiO_2-200 23
Figure S13. AC-TEM images and Pt NPs size distribution of Pt@NFM-SiO_2-300 24
Figure S14. AC-TEM images and Pt NPs size distribution of Pt@NFM-SiO_2-500

....25
Figure S15. AC-TEM images and Pt NPs size distribution of Pt@NFM-SiO_2-600 26
Figure S16. AC-TEM images and Pt NPs size distribution of Pt@NFM-SiO_2-700 27
Figure S17. AC-TEM images and Pt NPs size distribution of Pt@NFM-SiO_2-800 28
Figure S18. The mean sizes of Pt nanoparticles as a function of the pretreatment temperature. 29
Fig. S19. XPS spectra of Pt 4f over PtO_x@NFM-SiO_2 and Pt@NFM-SiO_2-X....30
Figure S20. The adsorption energy and corresponding adsorption Gipps free energy of O_2, H_2O, and HCHO molecules at each active site. 31
Figure S21. CO_2 selectivity as a function of time of NFM-SiO_2, PtO_x@NFM-SiO_2, and Pt@NFM-SiO_2-400...32
Figure S22. CO_2 selectivity as a function of time over Pt@NFM-SiO_2 at different reduction temperatures...33
Figure S23. FT-IR spectra of Pt@NFM-SiO_2 at different reduction temperatures. 34
Figure S24. XRD of Pt@NFM-SiO_2 under different reduction temperatures. 35
Figure S25. N_2 adsorption/desorption isotherms and pore size distribution curve of Pt@NFM-SiO_2-X and NFM-SiO_2. 36
Figure S26. CO_2 selectivity as a function of time over Pt@NFM-SiO_2-400, Pt/MCM-41-400, and Pt/SiO_2-400...37
Figure S27. HCHO oxidation over Pt/MCM-41-400, Pt/MCM-41-700, Pt/SiO_2-400, and Pt/SiO_2-700. 38
Figure S28. XRD of Pt/MCM-41-400, Pt/MCM-41-700, Pt/SiO_2-400, and Pt/SiO_2-700...39
Figure S29. XPS spectra of Pt 4f over Pt@NFM-SiO_2-400, Pt@NFM-SiO_2-400-used, Pt/MCM-41-400, Pt/MCM-41-400-used, Pt/SiO_2-400, and Pt/SiO_2-400-used. 40
Figure S30. H_2-TPR of Pt@NFM-SiO_2-400, Pt/MCM-41-400, and Pt/SiO_2-400. 41
Figure S31. O_2-TPO of Pt@NFM-SiO_2-400, Pt/MCM-41-400, and Pt/SiO_2-400. 42
Figure S32. EPR spectra of Pt@NFM-SiO_2-400, Pt/MCM-41-400, and Pt/SiO_2-400...43
Figure S33. O_2-DRIFTS spectra of Pt@NFM-SiO_2-400, Pt/MCM-41-400, and Pd/SiO_2-400 exposed in a 5% O_2 + Ar flow at 30 °C. Related dynamic evolution of the integrated area of surface reactive oxygen species. 44
Figure S34. H_2O-DRIFTS spectra of Pt@NFM-SiO_2-400, Pt/MCM-41-400, and Pd/SiO_2-400 exposed in a 5% O_2 + Ar flow at 30 °C. Related dynamic evolution of the integrated area of surface –OH species...45
Figure S35. HCHO conversion and CO_2 selectivity as a function of time over Pt@NFM-SiO_2-400 under different humidity...46

Figure S36. HCHO conversion and CO_2 selectivity as a function of time over Pt@NFM-SiO_2-400 at different concentration. 47
Figure S37. HCHO conversion and CO_2 selectivity as a function of time over Pt@NFM-SiO_2-400 under different WHSV 48
Figure S38. HCHO conversion and CO_2 selectivity as a function of time over Pt@NFM-SiO_2-400 under different temperature. 49
Figure S39. CO_2 selectivity of HCHO oxidation over Pt@NFM-SiO_2-400 for cycling test. 50

Experimental Procedures

Catalyst preparation

Synthesis of nano-flower-like mesoporous silica (NFM-SiO_2). Typically, 59.904 g cyclohexane, 3.84 mL n-amyl alcohol, and 6.3984 g TEOS were added to a 250 mL beaker and stir well, marked as liquid A. Then, 2.563 g cetyltrimethyl ammonium bromide (CTAB), 1.5366 g urea, and 76.8 mL H_2O were added to a 250 mL beaker and soaked in water at 40 °C until the solution was clarified, marked as liquid B. Subsequently, liquid B was slowly added to liquid A, stirred for 30 min, and transferred to a 200 mL hydrothermal reactor and oil bath at 120 °C for 6 h. After the hydrothermal reactor was cooled to room temperature, ethanol was added to demulsification. The initial sample was collected by centrifugation at 9000 rpm and washed three times with ethanol. After drying in an oven at 80 °C for 12 h and then calcining at 550 °C for 6 h, finally obtaining the NFM-SiO_2 sample.

Synthesis of closed mesoporous silica (MCM-41). First, 1.15g of CTAB was added to 153.75 mL of water, then 312.5 mL of ethanol and 13.5 mL of ammonium hydroxide were added and stirred for 30 min. Then, 4.75 g TEOS was added to the above solution, stirred at high speed for 10 min, and aged at room temperature for 24 h. Subsequently, it was centrifuged at 9000 rpm and washed 3-5 times with deionized water. Finally, drying in an oven at 80 °C for 12 h and calcining at 550 °C for 6 h to obtain MCM-41.

Synthesis of silica spheres (SiO_2). Add 10 mL ammonium hydroxide, 100 mL ethanol, and 10 mL H_2O into a 250 mL beaker, stir at high speed for 1 h, then add 5 mL TEOS and stir for 20 h. Subsequently, centrifugal separation was performed at 9000 rpm and washed 3-5 times with deionized water. Finally, drying in an oven at 80 °C for 12 h and calcining at 550 °C for 6 h to obtain SiO_2.

Synthesis of Pt@NFM-SiO_2, Pt/MCM-41, and Pt/SiO_2. The Pt@NFM-SiO_2 catalyst was prepared by a facile wetness impregnation method. Briefly, 0.5 g NFM-SiO_2, a certain amount of $Pt(NH_3)_4(NO_3)_2$, and 4 mL H_2O were added to a 10 mL beaker.

Subsequently, it was stirred for 4 h, and then H_2O was removed in a water bath at 80 °C. Finally, dried in an oven at 80 °C for 12 h and reduced by 10% H_2/Ar for 3 h to obtain the Pt@NFM-SiO_2-X sample, where X represents the reduction temperature. The reduction temperatures used in the experiment were 200, 300, 400, 500, 600, 700, and 800 °C. The preparation method of Pt/MCM-41 and Pt/SiO_2 samples is the same as that of Pt@NFM-SiO_2, only the support materials are changed.

Catalyst characterization

Powder X-ray diffraction (XRD) patterns were obtained from an X-ray diffractometer (UltimaIV, Rigaku) that operated at 40 kV and 30 mA with a Cu target and Kα-ray irradiation (λ = 1.54178 Å). The Pt content of samples was determined by inductively coupled plasma optical emission spectrometry (ICP-OES), which was performed on an Avio 500 ICP OES with Dichroic Spectral Combiner (DSC) technology (Perkin Elmer, Singapore). Fourier transform infrared spectra (FT-IR) were recorded on a spectrometer (Thermo Fisher Nicolet iS10 FT-IR, transmission mode) with a scanning range of 650-4000 cm^{-1}. The specific surface area (S_{BET}) and pore structure of the samples were measured by a physisorption analyzer using N_2 adsorption–desorption at -196 °C (TriStar II 3020 M, Micromeritics, USA). Before N_2 adsorption, the samples were degassed at 300 °C for 300 min to remove the physisorbed moisture. The size and morphology of the samples were characterized through a high-resolution transmission electron microscope (HRTEM, FEI Tecnai G2 F20) and scanning electron microscope (SEM, Gemini 500). The high-angle annular dark field spherical aberration-corrected STEM (AC-HAADF-STEM) images were obtained using a JEM-ARM200P microscope with an aberration corrector for a pro-forming system, operated at 200 kV. X-ray photoelectron spectroscopy (XPS) measurements were performed with an ESCALab250 photoelectron spectroscope. Temperature programmed reduction (TPR) experiments were carried out in a temperature-programmed system (Builder, PCA-1200, China) equipped with a thermal conductivity

detector (TCD) coupled with an online mass spectrometer (HPR20, Hiden, UK). For each experiment, 60 mg of sample were loaded into a U-shaped quartz reactor supported over a quartz wool bed. Subsequently, the sample was pretreated in ultra-high purity Ar at 400 °C for 60 min. After cooling to room temperature, the gas was switched to a 10% H_2/Ar (30 mL min^{-1}) and the temperature was increased up to 900 °C at a ramp rate of 10 °C min^{-1} while monitoring hydrogen consumption with a TCD detector. O_2-temperature-programmed oxidation (O_2-TPO) was conducted using a PCA-1200 apparatus to study the activation capacity of gaseous oxygen over Pt@NFM-SiO_2-400, Pt/MCM-41-400, and Pt/SiO_2-400. Typically, 60 mg of sample was placed in a quartz reactor and pretreated in Ar flow at 400 °C for 60 min, and then cooling to room temperature. Subsequently, the gas was switched to a 5% O_2/Ar (30 mL min^{-1}) and the temperature was increased up to 900 °C at a ramp rate of 10 °C min^{-1}, while monitoring hydrogen consumption with a TCD detector.

The X-ray absorption spectra were collected on the beamline BL01C1 in the National Synchrotron Radiation Research Center (NSRRC), with electron energy of 1.5 GeV and a beam current between 100 and 200 mA. The radiation was monochromatized by a Si (111) double-crystal monochromator. The XAFS data were recorded in fluorescence mode with standard Lytle ion chambers for the Pd K-edge. The EXAFS fitting was applied through Athena and Artemis software, and Wavelet transformation (WT) was also employed using the software package developed by Funke and Chukalina using the Morlet wavelet with $\kappa = 10$, $\sigma = 1$.[1-3]

Activity test

Formaldehyde (HCHO) oxidation was performed in a continuous-flow fixed-bed reactor (inner diameter 8 mm). Gaseous formaldehyde was generated by flowing dry air through paraformaldehyde, which was placed in a round-bottomed flask in a water bath. Subsequently, the activity was evaluated at room temperature under 120 mL min^{-1} 100 ppm HCHO/air. The weight hourly space velocity (WHSV) and relative humidity

(RH) were fixed at 120,000 mL $g_{cat.}^{-1}h^{-1}$ and 40%, respectively. The HCHO and CO_2 concentrations were measured by a multigas analyzer (Gasera One, Gasera Ltd., Finland). The RH was tested by a hygrometer (Model: A2000-ES; Range: T = -40~85 °C, RH= 0~100%) from Shenzhen Huato System Co., LTD. The HCHO conversion (X_{HCHO}) and CO_2 selectivity (S_{CO_2}) were calculated according to the following equations:

$$X_{HCHO} = \frac{[HCHO]_{in} - [HCHO]_{out}}{[HCHO]_{in}} \times 100\% \quad (1)$$

$$S_{CO_2} = \frac{[CO_2]_{out}}{[C]_{out,total}} \times 100\% \quad (2)$$

where $[HCHO]_{in}$ and $[HCHO]_{out}$ are noted as the inlet and outlet HCHO concentrations in the feed stream, respectively. The $[CO_2]_{out}$ and $[C]_{out,total}$ represent the outlet CO_2 concentrations, and the total carbon concentrations present in the combustion products, respectively.

The study of the humidity effect and the stability test were applied with the same test methods.

Reaction kinetics measurement

The kinetic data for HCHO oxidation of Pt@NFM-SiO_2-400, Pt/MCM-41-400, and Pt/SiO_2-400 were tested in a fixed-bed reactor (101.325 kPa) according to previous reported literature.[4] Typically, the feed gas were consisted of 100 ppm HCHO/air with a WHSV of 4800,000 mL $g_{cat.}^{-1}h^{-1}$ and 3 mg of catalyst mixed with 47 mg of inert quartz sand (40-60 mesh) was used for each testing while the internal and external diffusion have been eliminated. The HCHO conversions were controlled within 15%.

The r_{HCHO} and r_{HCHO} (mol $(g_{cat}\cdot s)^{-1}$) expressed the reaction rate were calculated by the equation of $r_{HCHO} = X_{HCHO} \cdot V_{HCHO}/g_{cat}$, where X_{HCHO} is the conversion, V_{HCHO} and is the gas flow rate (mol s^{-1}) of HCHO, g_{cat} is the weight of the catalyst.

The total turnover frequency (TOF) (s^{-1}) of HCHO was calculated by the equation of $TOF_{total} = X_{HCHO} \cdot V_{HCHO} \cdot N_A/N_{total}$, where N_A is Avogadro constant and N_{total} is total atom numbers of Pt, which can be calculated by the equation of $N_{total} =$

$(m_{pt}/M_{pt}) \cdot N_A$, where m_{pt} is the mass and M_{pt} is relative atomic mass of Pt.

Density functional theory calculations

The Vienna ab initio simulation package (VASP)[5,6] was applied to perform all density functional theory (DFT) calculations within the generalized gradient approximation (GGA) using the Perdew-Burke-Ernzerhof (PBE)[7] formulation. The projected augmented wave (PAW) potentials[8] were chosen to describe the ionic cores and take valence electrons into account using a plane wave basis set with a kinetic energy cutoff of 500 eV. Partial occupancies of the Kohn-Sham orbitals were allowed using the Gaussian smearing method and a width of 0.1 eV. The electronic energy was considered self-consistent when the energy change was smaller than 10^{-5} eV. A geometry optimization was considered convergent when the force change was smaller than 0.05 eV/Å. Grimme's DFT-D3 methodology was used to describe the dispersion interactions.[9] During structural optimizations, the Γ point in the Brillouin zone was used for k-point sampling, and SiO_2 atoms were allowed to be fixed. Finally, the adsorption energies (E_{ads}) were calculated as $E_{ads} = E_{ad/sub} - E_{ad} - E_{sub}$, where $E_{ad/sub}$, E_{ad}, and E_{sub} are the total energies of the optimized adsorbate/substrate system, the adsorbate in the structure, and the clean substrate, respectively. The free energy of a gas phase molecule or an adsorbate on the surface was calculated by the equation $G = E + E_{ZPE} - TS$, where E is the total energy, E_{ZPE} is the zero-point energy, T is the temperature in kelvin (298.15 K is set here), and S is the entropy.

In situ DRIFTS measuring processes

CO-DRIFTS adsorption

In situ CO-DRIFTS adsorption spectra was recorded on Thermo Fisher Nicolet iS10 FT-IR equipped with MCT detector at room temperature. In the DRIFT cell with KBr windows connected with a gas flow system, the sample was pretreated at 300 °C in N_2 for 30 min and then cooled to 25 °C in N_2. After the background spectra were recorded

at the temperature, N_2 gas was replaced by 2% CO/N_2 (30 mL min^{-1}) flow for 30 min and N_2 purge for 10 min.

O_2-DRIFTS adsorption

The O_2-DRIFTS adsorption spectra was performed on Thermo Fisher Nicolet iS10 FT-IR equipped with MCT detector at 30 °C. The sample was pretreated at 300 °C in N_2 for 30 min and then cooled to 30 °C in N_2. After the background spectra were recorded at 30 °C, N_2 gas was replaced by 5% O_2/Ar (30 mL min^{-1}) flow for 30 min.

H_2O-DRIFTS adsorption

The H_2O-DRIFTS adsorption spectra was performed on the same Thermo Fisher Nicolet iS10 FT-IR equipped with MCT detector at 30 °C. The sample was pretreated at 300 °C in N_2 for 30 min and then cooled to 30 °C in N_2. After the background spectra were recorded at 30 °C, N_2 gas was replaced by H_2O/N_2 (30 mL min^{-1}, RH=40%) flow for 30 min.

HCHO in situ DRIFTS adsorption and oxidation at 25 °C

The Pt@NFM-SiO_2-400 sample was pretreated at 300 °C in N_2 for 30 min and then cooled to 25 °C in N_2. After the background spectra were recorded at the temperature, N_2 gas was replaced by 100 ppm $HCHO/N_2$ or 100 ppm $HCHO/O_2/N_2$, 100 ppm $HCHO/H_2O/N_2$, and 100 ppm $HCHO/O_2/H_2O/N_2$ (100 mL min^{-1}, RH = 40%) flow for 30 min and the spectra were recorded.

Supplementary Tables

Table S1. Physicochemical properties and Pt content of catalysts measured by N_2 sorption isotherms and ICP.

Samples	S_{BET} ($m^2\ g^{-1}$)[a]	D_{meso} (nm)[b]	Pt content (wt.%)[c]
SiO_2	40	-	-
MCM-41	1147	2.1	-
NFM-SiO_2	492	3.4	-
Pt/SiO_2-400	29	2.1	0.13
Pt/MCM-41-400	959	2.0	0.12
Pt@NFM-SiO_2-200	470	3.4	-
Pt@NFM-SiO_2-300	467	3.4	-
Pt@NFM-SiO_2-400	465	3.4	0.13
Pt@NFM-SiO_2-500	460	3.4	-
Pt@NFM-SiO_2-600	445	3.4	-
Pt@NFM-SiO_2-700	347	3.4	-
Pt@NFM-SiO_2-800	304	3.4	0.14

[a] Calculated by BET method.

[b] Determined by BJH method.

[c] Obtained from ICP results.

Table S2. EXAFS fitting parameters at the Pt L3-edge for various samples（S_0^2=0.86）.

Sample	shell	$^a CN$	$^b R$(Å)	$^c \sigma^2$	$^d \Delta E_0$	R factor
Pt foil	Pt-Pt	12	2.76 ± 0.01	0.0048	7.9 ± 0.4	0.0023
	Pt-O	6.1 ± 0.3	2.02 ± 0.01	0.0026		
PtO_2	Pt-Pt	9.2 ± 0.9	3.11 ± 0.01	0.0050	10.1 ± 0.7	0.0071
	Pt-O1	7.1 ± 1.3	3.65 ± 0.02	0.0012		
	Pt-O	1.1 ± 0.1	1.98 ± 0.01	0.0012		
Pt@NFM-SiO_2-200	Pt-Si	0.7 ± 0.1	2.37 ± 0.02	0.0064	8.5 ± 1.4	0.0019
	Pt-Pt	3.7 ± 0.1	2.75 ± 0.01	0.0044		
	Pt-O	1.2 ± 0.6	2.01 ± 0.05	0.0174		
Pt@NFM-SiO_2-400	Pt-Si	0.6 ± 0.2	2.35 ± 0.02	0.0016	8.1 ± 1.9	0.0099
	Pt-Pt	4.8 ± 0.3	2.75 ± 0.01	0.0028		

^{a} *CN*: coordination numbers; ^{b} *R*: bond distance; ^{c} σ^2: Debye-Waller factors; ^{d} ΔE_0: the inner potential correction. *R* factor: goodness of fit.

Table S3. Kinetic catalytic performances of Pt@NFM-SiO_2-400 and related catalysts.[a]

Samples	Ea (kJ mol^{-1})	Rate$^{@70℃}$ × 10^7 (mol $g_{cat.}^{-1} s^{-1}$)	TOF$^{@70℃}$ × 10^2 (s^{-1})
Pt@NFM-SiO_2-400	21.4	1.93	10.3
Pt/MCM-41-400	22.2	0.88	3.6
Pt/SiO_2-400	33.9	0.26	2.0

[a] Kinetic test conditions: The kinetics data for HCHO oxidation was tested in a fixed-bed reactor at atmospheric pressure. The feed gas was consisted of 100 ppm of HCHO, 40% RH, air, WHSV 4800, 000 mL $g_{cat.}^{-1}$ h^{-1}, and 3 mg of catalyst diluted with 47 mg of inert quartz sand (40-60 mesh) was used for each testing, in which the internal diffusion and external diffusion have been eliminated. The HCHO conversion was controlled to below 15%.

Table S4. Comparison with literatures about the performances of Pt based catalysts for HCHO oxidation.

Catalysts	Pt wt.%	Reaction conditions			Conversion (%)	Stability (h)	References
		HCHO (ppm)	T (°C)	WHSV (mL g^{-1} h^{-1})			
**Pt@NFM-SiO_2**	**0.13**	**100**	25	**120000**	**98**	**56**	**This work**
Pt/MnO_2-BN20	2.85	200	25	-	~96	-	Angew. Chem. Int. Ed. 2021, 60, 6377-6381
1.5%-Pt/SBT-400	1.5	100	25	-	~90	-	Environ. Sci. Technol. 2019, 53, 3610-3619
Pt/Fe_2O_3-HO	1	90	25	160000	~80	24	J. Hazard. Mater., 2020, 395, 122628
Pt/TiO_2	0.92	120	25	600000	~100	-	ACS Catal. 2022, 12, 5565-5573
Pt/γ-Al_2O_3	0.2	120	25	120000	~100	50	Appl. Catal. B Environ. Energy 2024, 357, 124342
Pt/$H_2Ti_2O_5$-TiO_2	0.49	120	25	800000	~97	-	Appl. Catal. B Environ. Energy 2025, 363, 124813
Pt/SiO_2-4.5	0.19	100	25	60000	~98	48	Appl. Catal. B Environ. Energy. 2023, 331, 122672

Note: Currently, Pt-based catalysts show excellent catalytic performance in HCHO oxidation and have been widely studied. However, it remains a significant challenge to study the role of various Pt sites at the atomic level in HCHO oxidation, and no relevant reports have been reported. In this study, we construct a sub-nanometric Pt ensemble catalyst with multi-type active sites via a dual-confinement strategy. Meanwhile, we determine the type of Pt sites by in situ characterization methods and study the role of each Pt site in the activation of O=O, H−OH, and C−H bonds by DFT calculations. The result shows that Pt top sites predominantly serve as pivotal centers for O=O bond activation, whereas Pt−O−Si interfacial sites primarily govern the activation of H−OH and C−H bonds. As shown in **Table S4**, we compared the oxidation performance for HCHO oxidation of a series of oxide-supported Pt catalysts and no further element doping modification. It can be seen that Pt@NFM-SiO_2 shows excellent catalytic

activity and stability for HCHO oxidation over the Pt-based catalysts. Specifically, the Pt loading is only ~0.13 wt%, which can remain ~98% conversion of HCHO oxidation within 56 hours. Our study not only fills the gap in the current research on HCHO oxidation but also inspires innovative approaches for the rational design of efficient heterogeneous catalysts.

Table S5. Summary of the vibrational modes and positions of surface species in the oxy-carbon surface species region (1000–4000 cm^{-1}).

Species	Band position (cm^{-1})		Reference
	This work	Literature range	
Surface -OH	3662	3414-3730	[10]
DOM	1028,1031,1035,1172,1173,1176, 1180,1300,1303,1434	1061-2977	[10-13]
$HCOO^-$	1624	1358-2988	[10-13]
CO	2058,2068,2072	1931-2120	[14,15]
HCHO	1719,1723	1700-1750	[16]
H_2O	3218,3228,3229,3320	3000-3330	[10]

Supplementary Figures

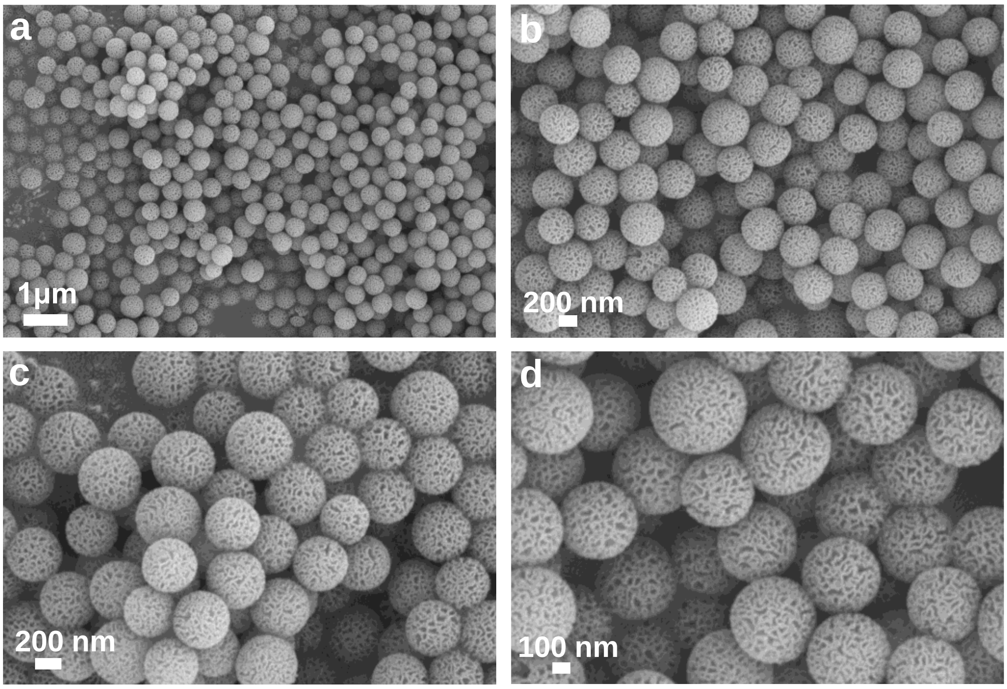

Figure S1. SEM images of NFM-SiO_2.

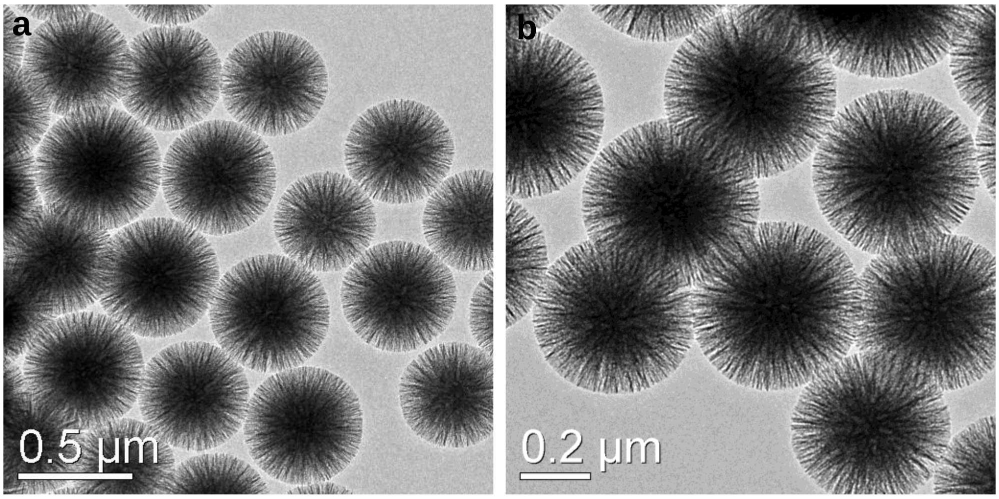

Figure S2. TEM images of NFM-SiO_2.

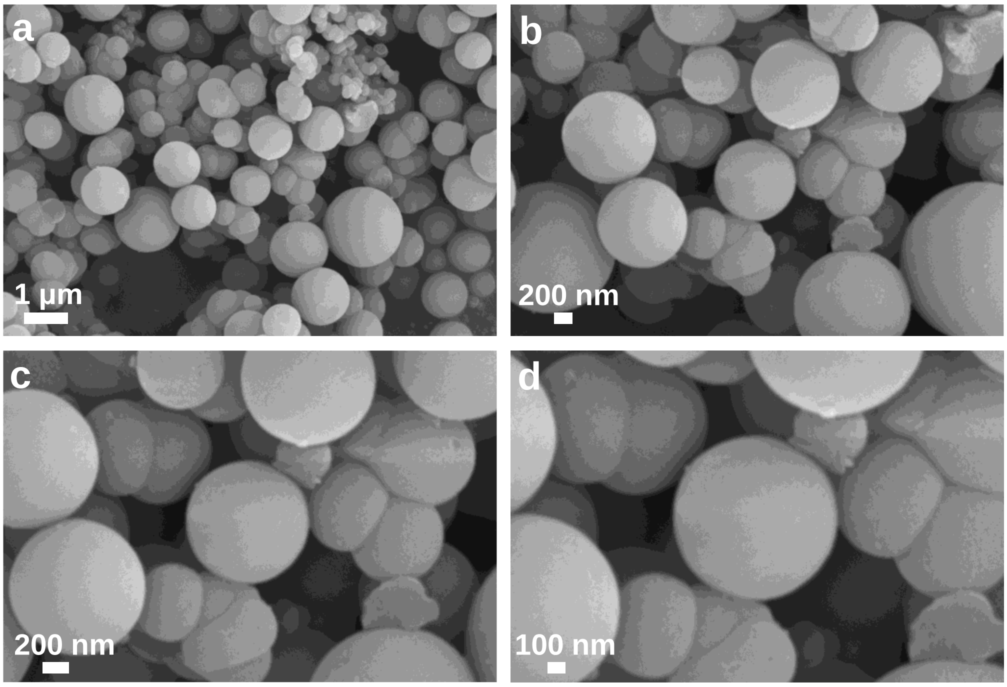

Figure S3. SEM images of Pt/MCM-41-400.

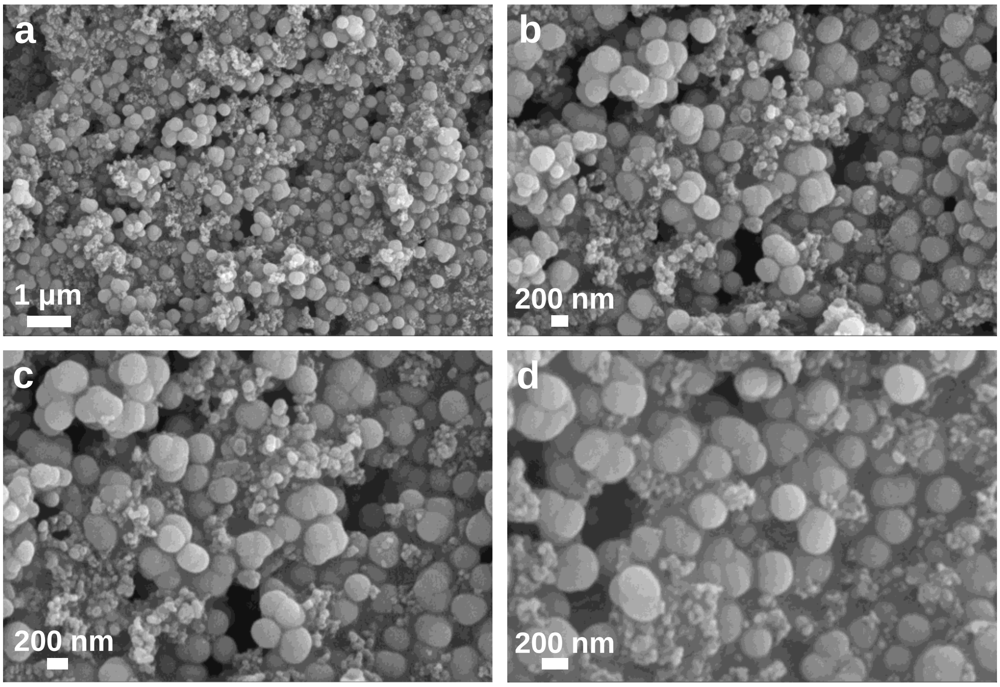

Figure S4. SEM images of Pt/SiO_2-400.

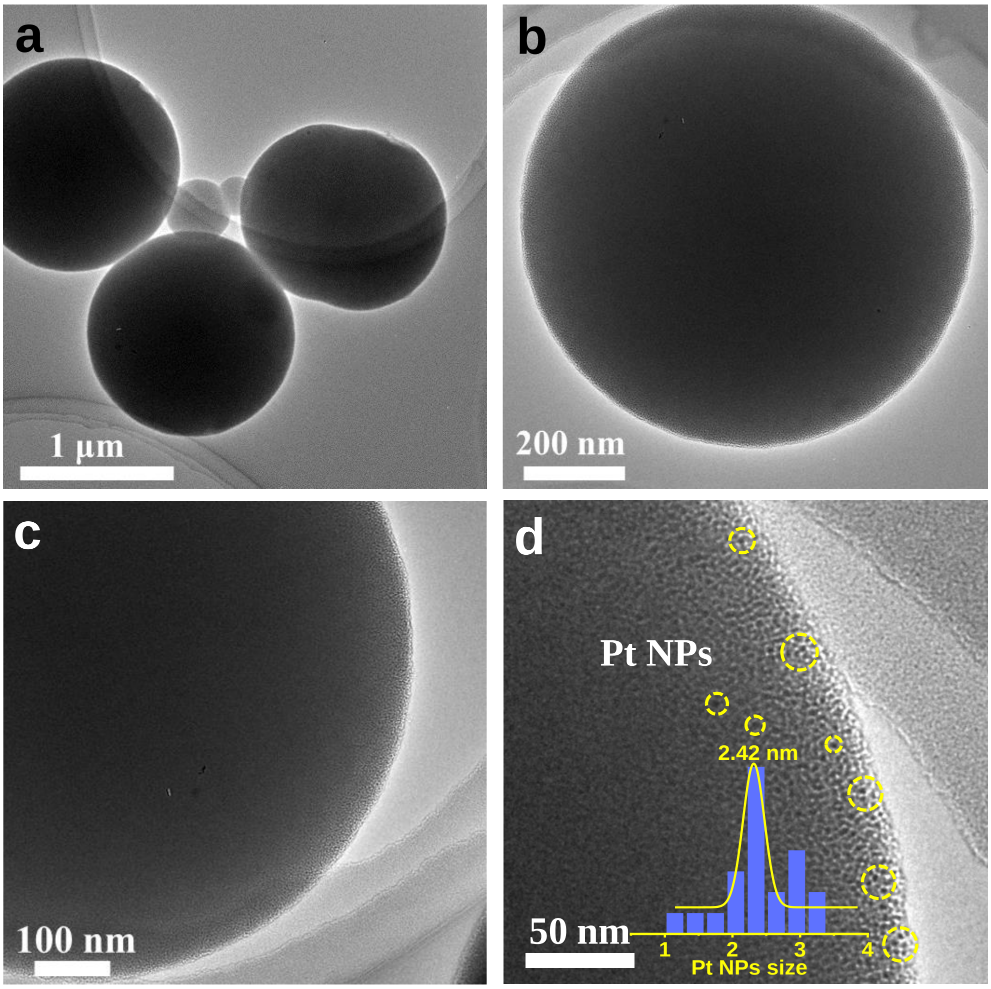

Figure S5. TEM images and Pt NPs size distribution of Pt/MCM-41-400.

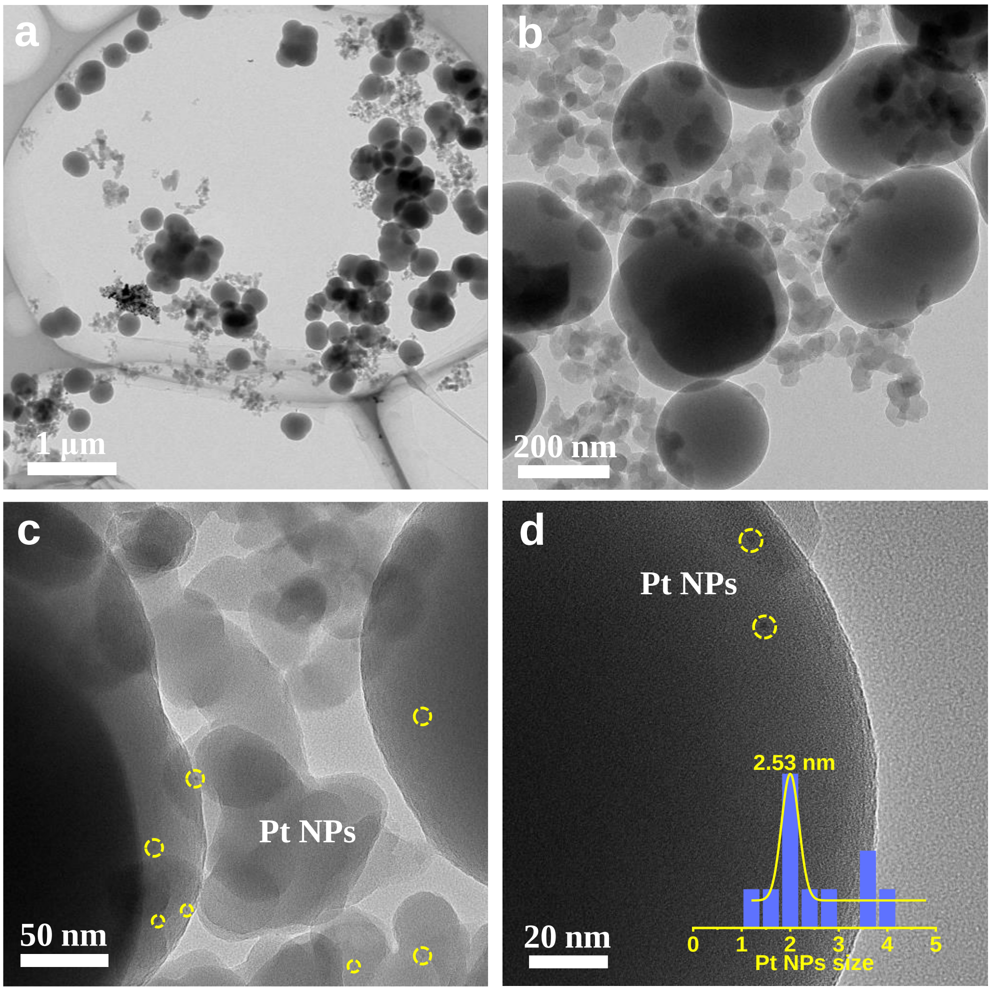

Figure S6. TEM images and Pt NPs size distribution of Pt/SiO_2-400.

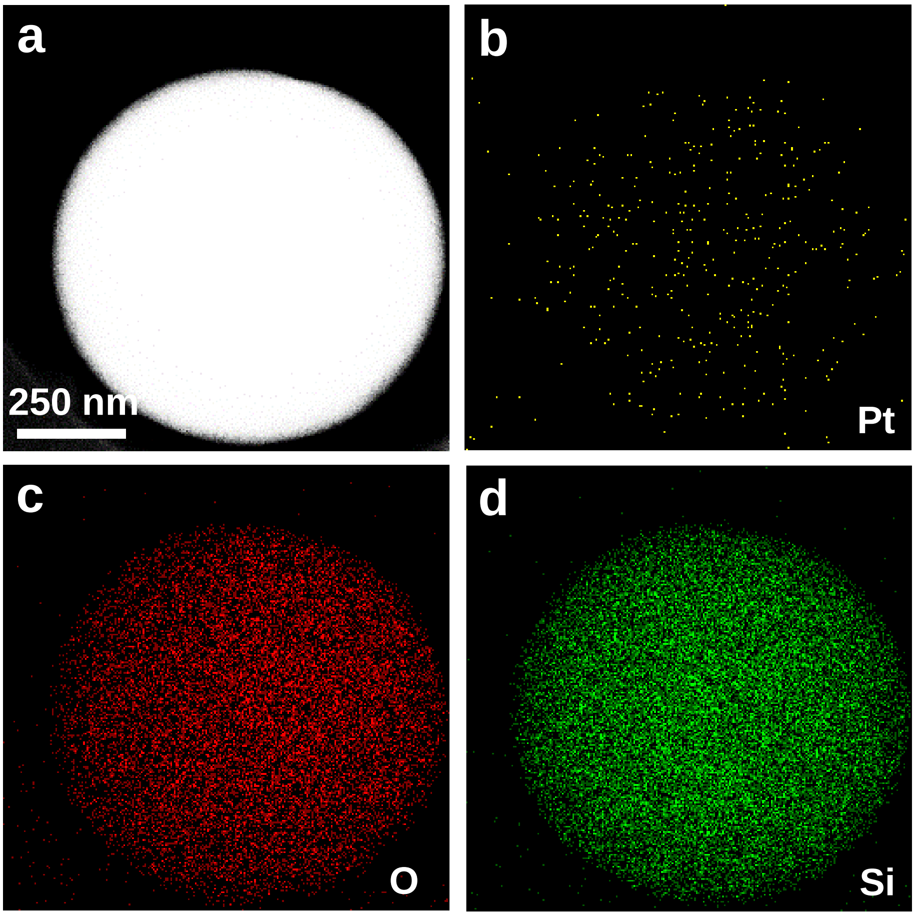

Figure S7. a) HAADF-STEM and b-d) EDS-Mapping of Pt/MCM-41-400.

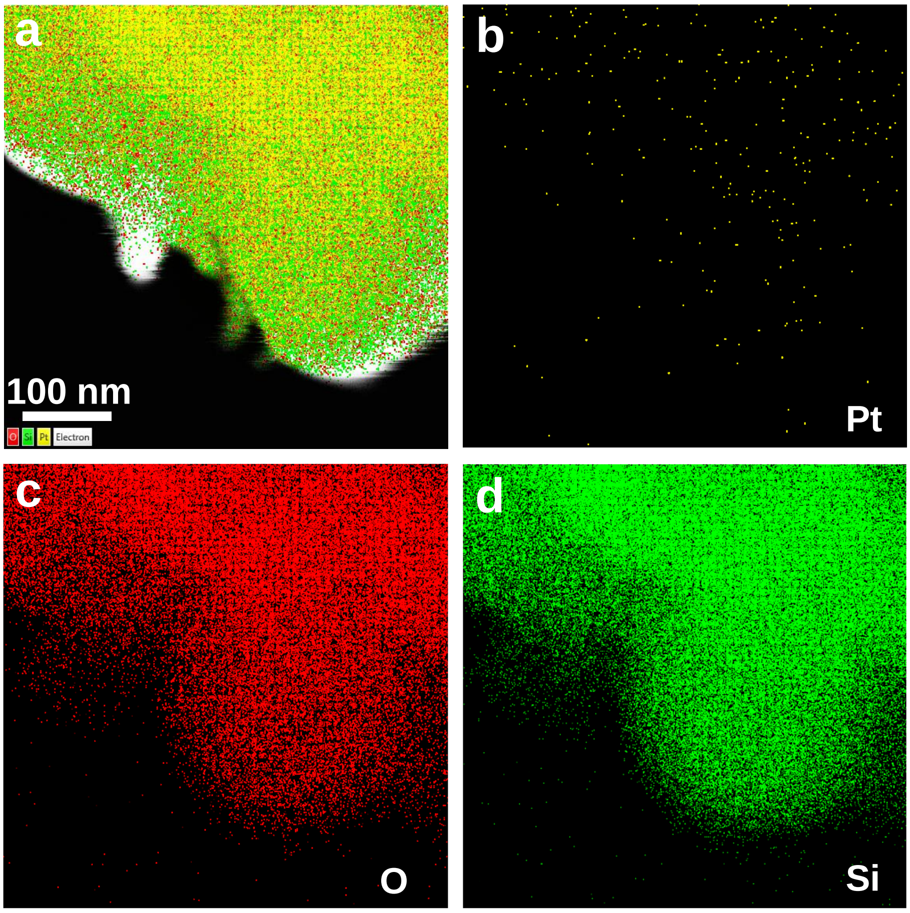

Figure S8. EDS-Mapping of Pt/SiO_2-400.

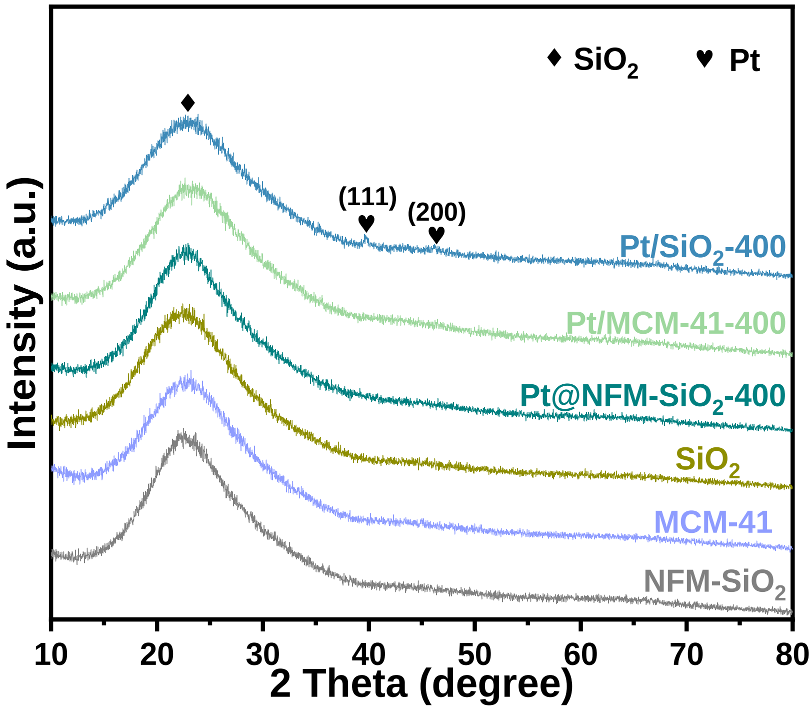

Figure S9. XRD patterns of Pt@NFM-SiO_2-400 and related catalysts.

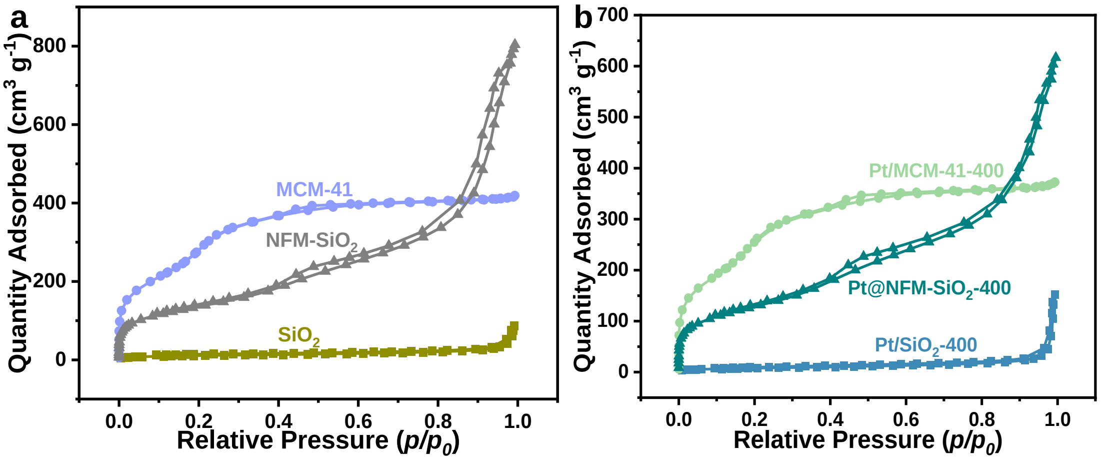

Figure S10. N_2 adsorption/desorption isotherms of Pt@NFM-SiO_2-400 and related catalysts.

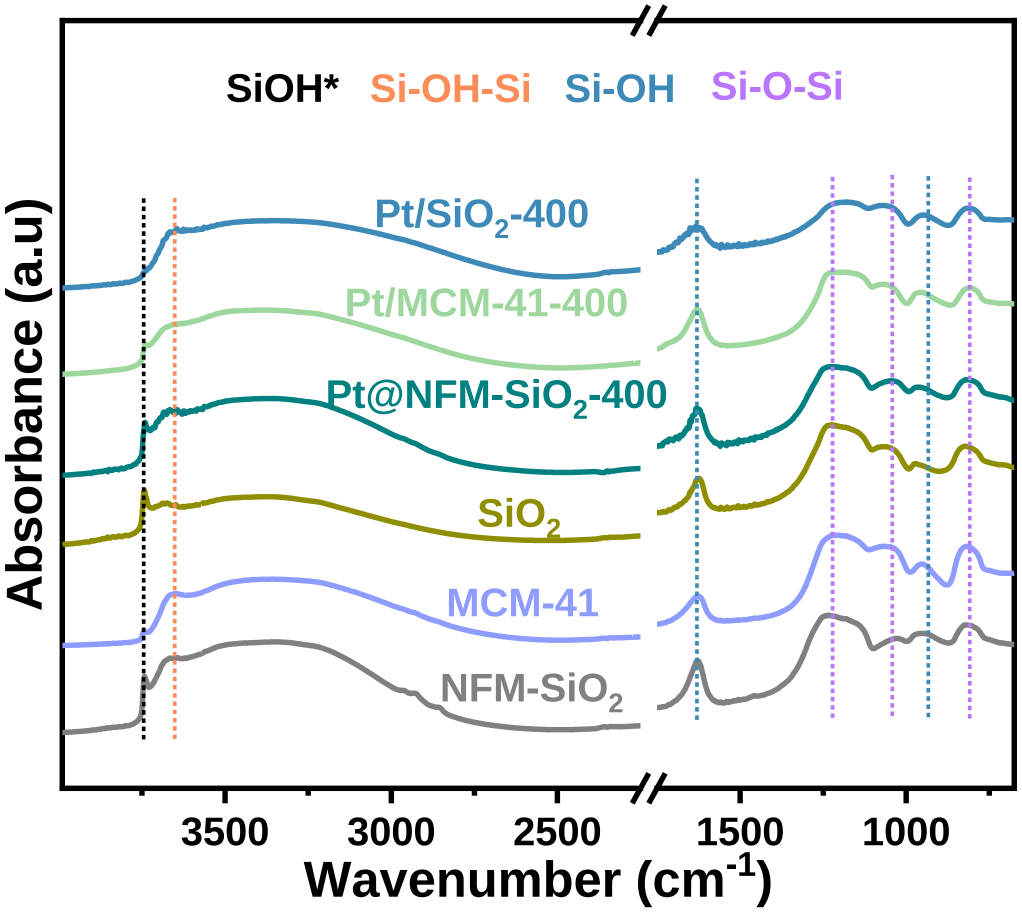

Figure S11. FT-IR spectra of Pt@NFM-SiO_2-400 and related catalysts.

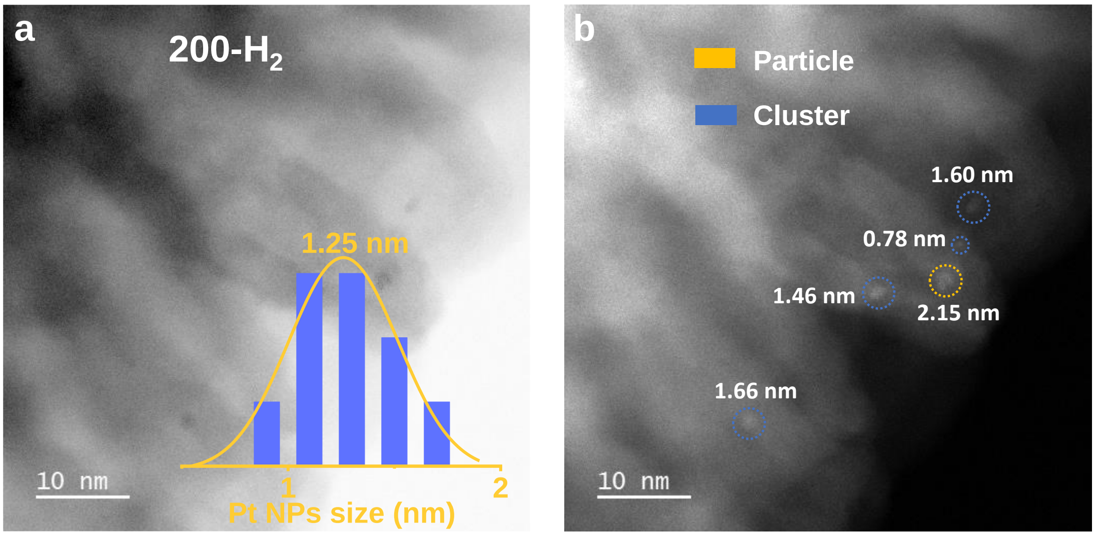

Figure S12. AC-TEM images and Pt NPs size distribution of Pt@NFM-SiO_2-200.

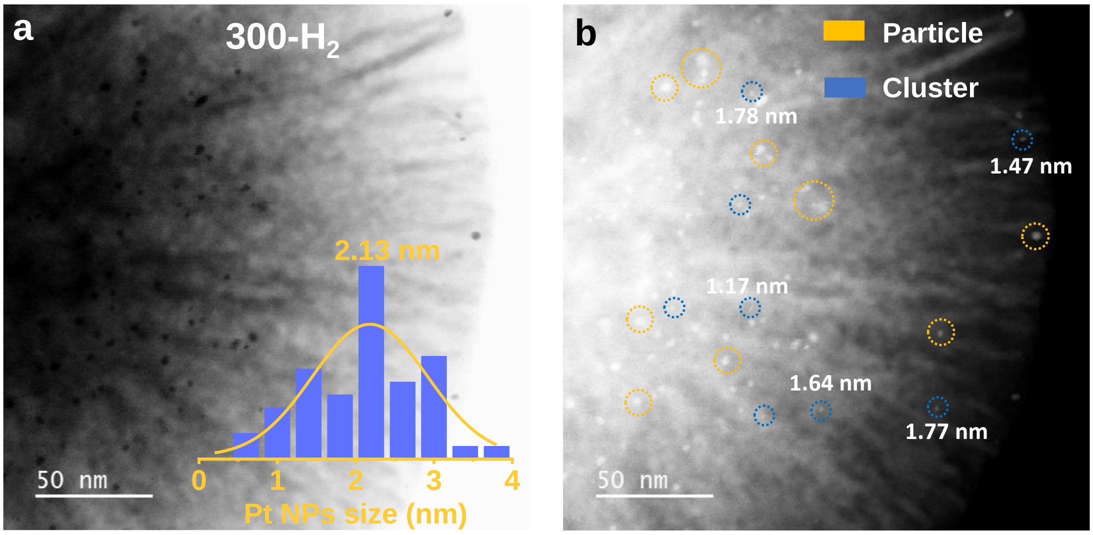

Figure S13. AC-TEM images and Pt NPs size distribution of Pt@NFM-SiO_2-300.

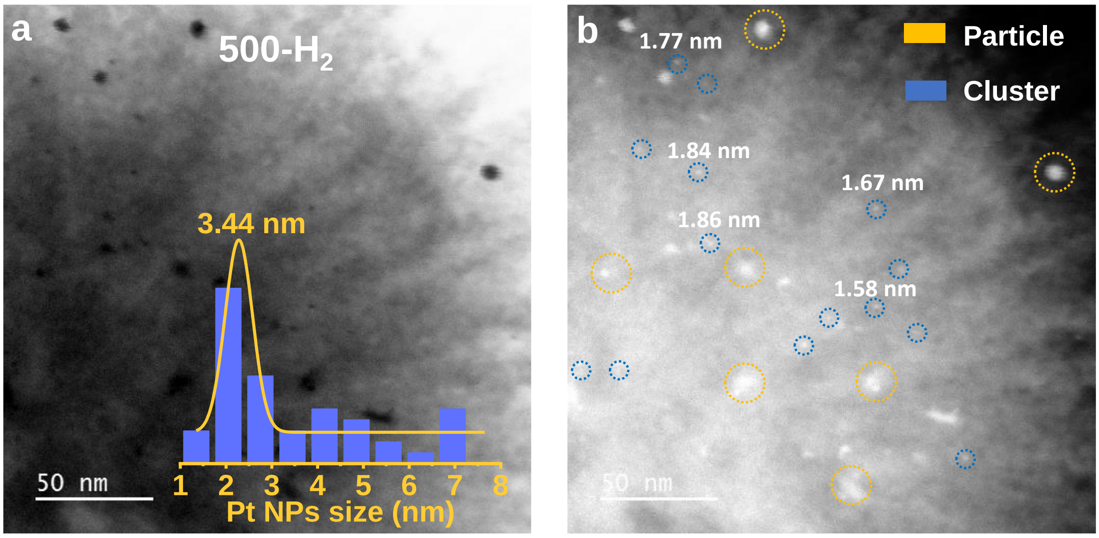

Figure S14. AC-TEM images and Pt NPs size distribution of Pt@NFM-SiO_2-500.

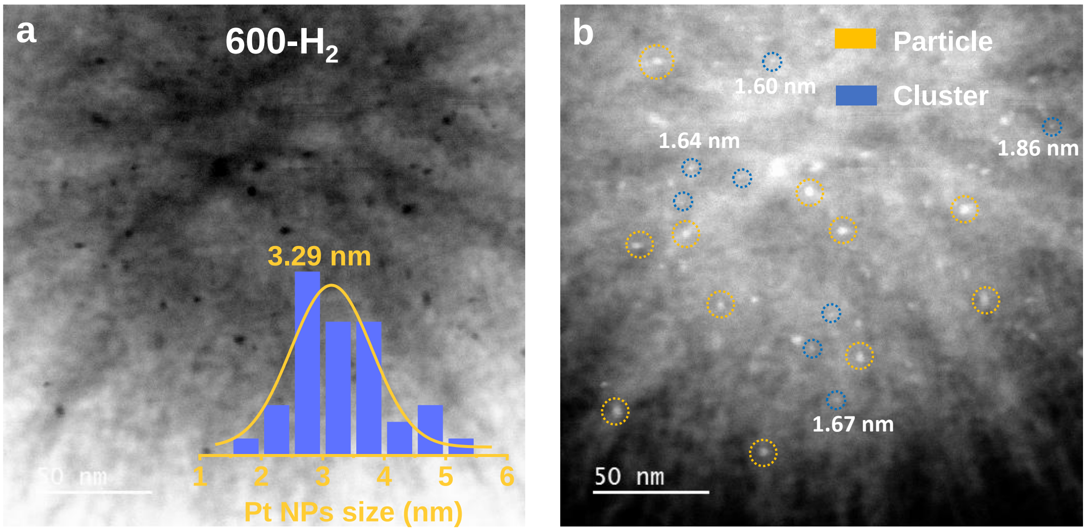

Figure S15. AC-TEM images and Pt NPs size distribution of Pt@NFM-SiO_2-600.

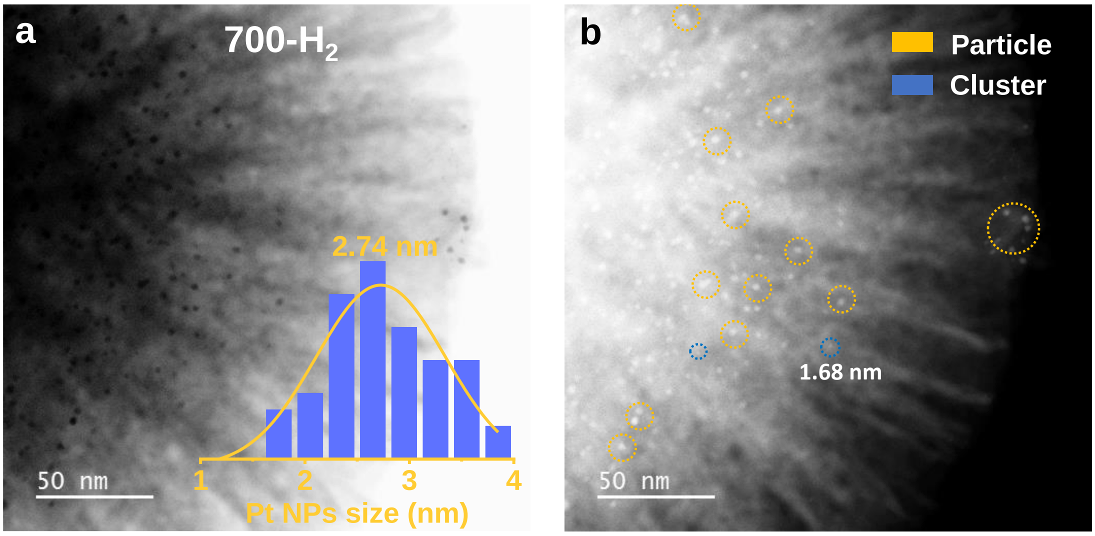

Figure S16. AC-TEM images and Pt NPs size distribution of Pt@NFM-SiO_2-700.

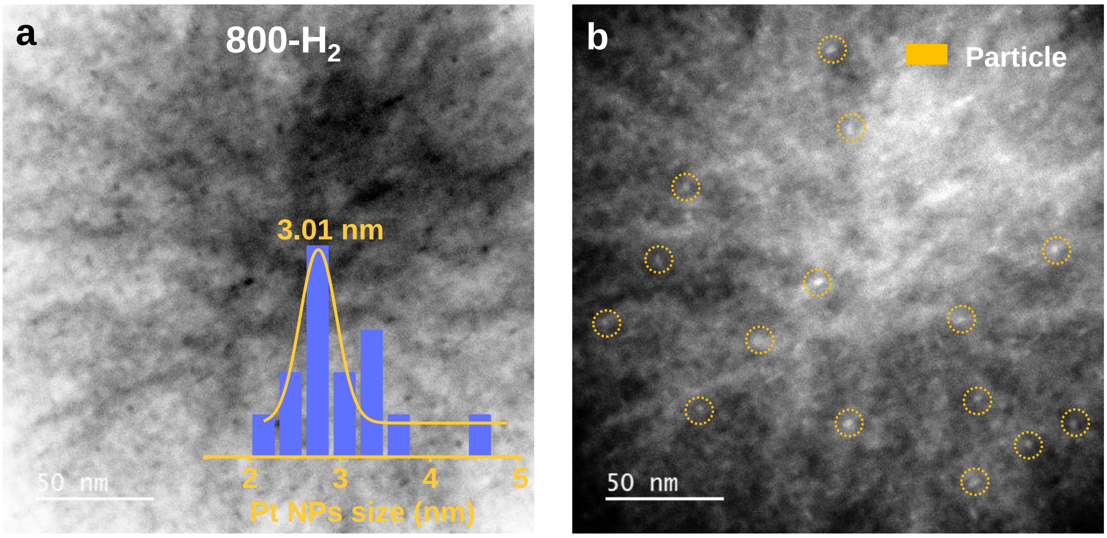

Figure S17. AC-TEM images and Pt NPs size distribution of Pt@NFM-SiO_2-800.

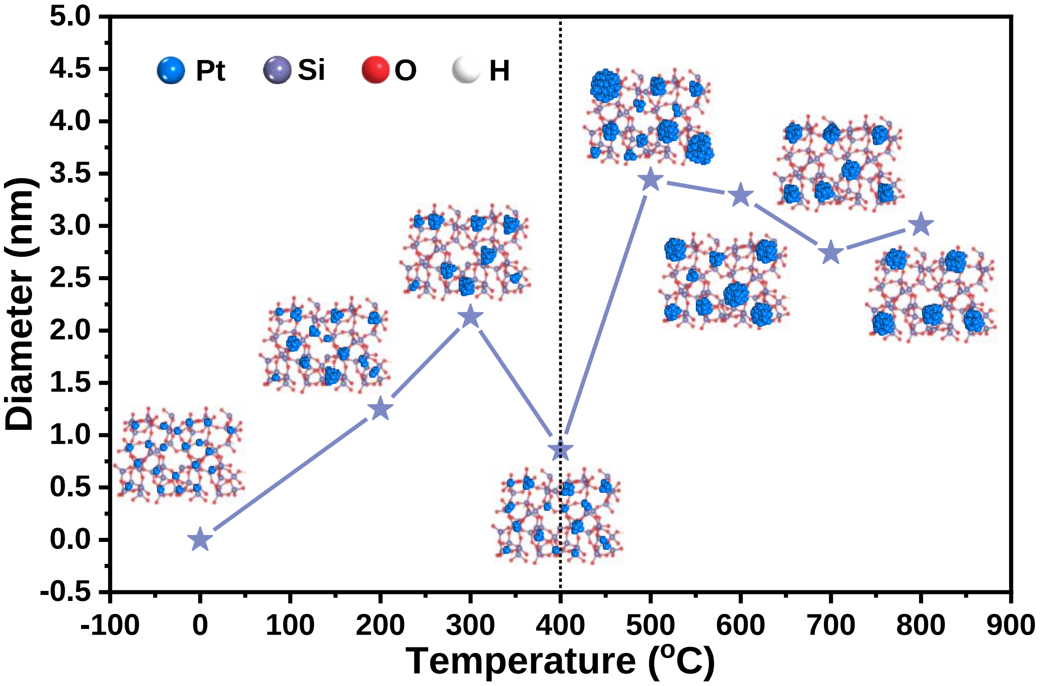

Figure S18. The mean sizes of Pt nanoparticles as a function of the pretreatment temperature.

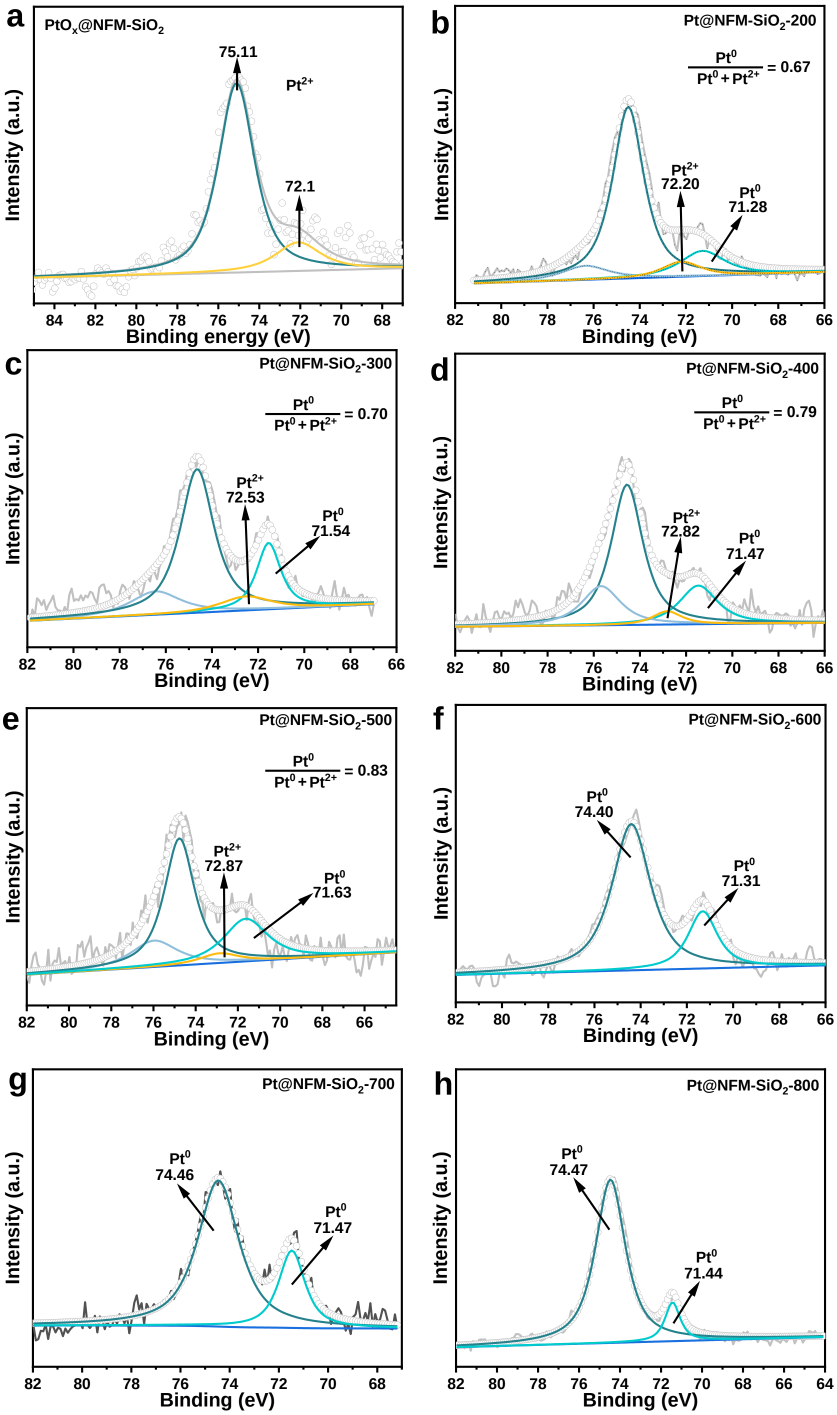

Fig. S19. XPS spectra of Pt 4f over PtO_x@NFM-SiO_2 and Pt@NFM-SiO_2-X (X is the reduction temperatures).

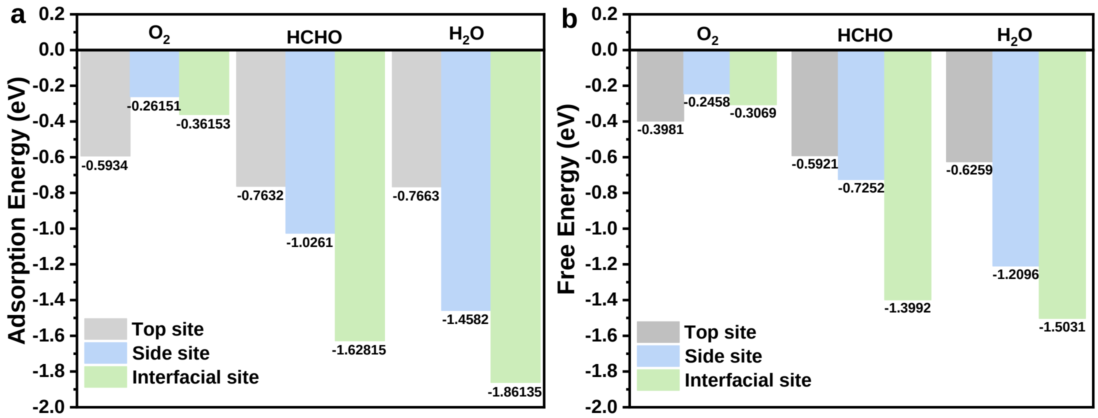

Figure S20. a) The adsorption energy and b) corresponding adsorption Gipps free energy of O_2, H_2O, and HCHO molecules at each active site.

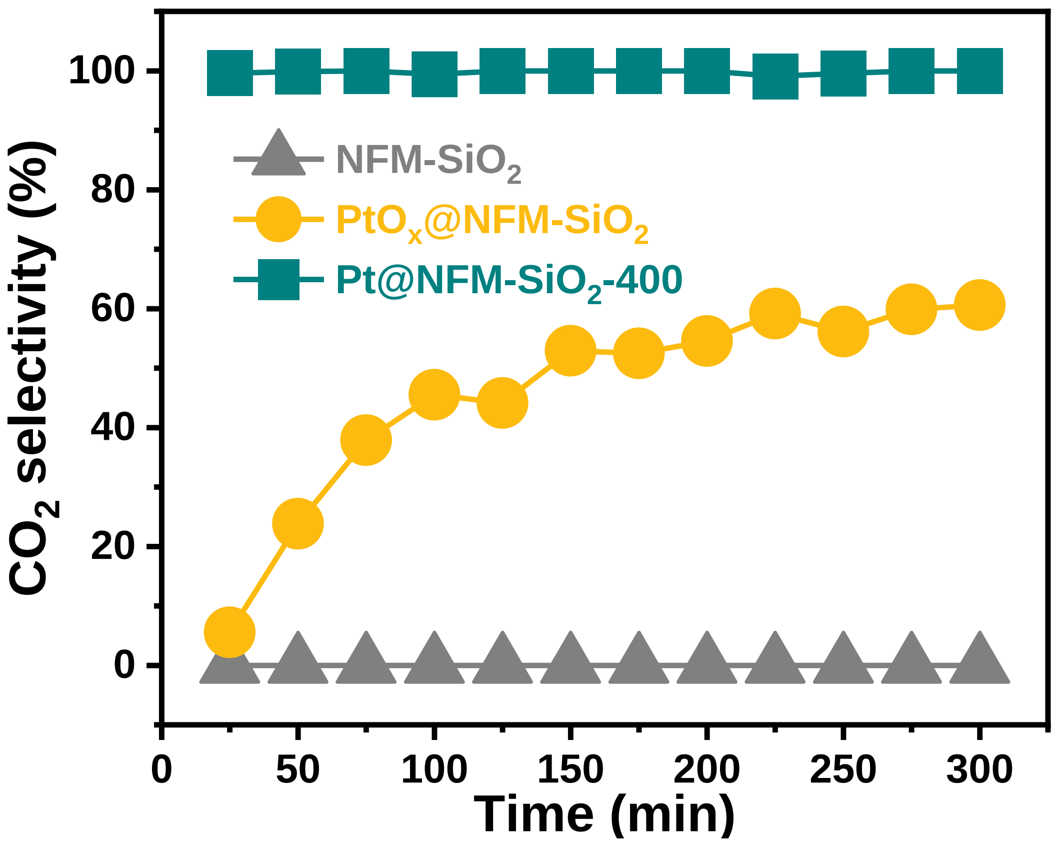

Figure S21. CO_2 selectivity as a function of time of NFM-SiO_2, PtO_x@NFM-SiO_2, and Pt@NFM-SiO_2-400.

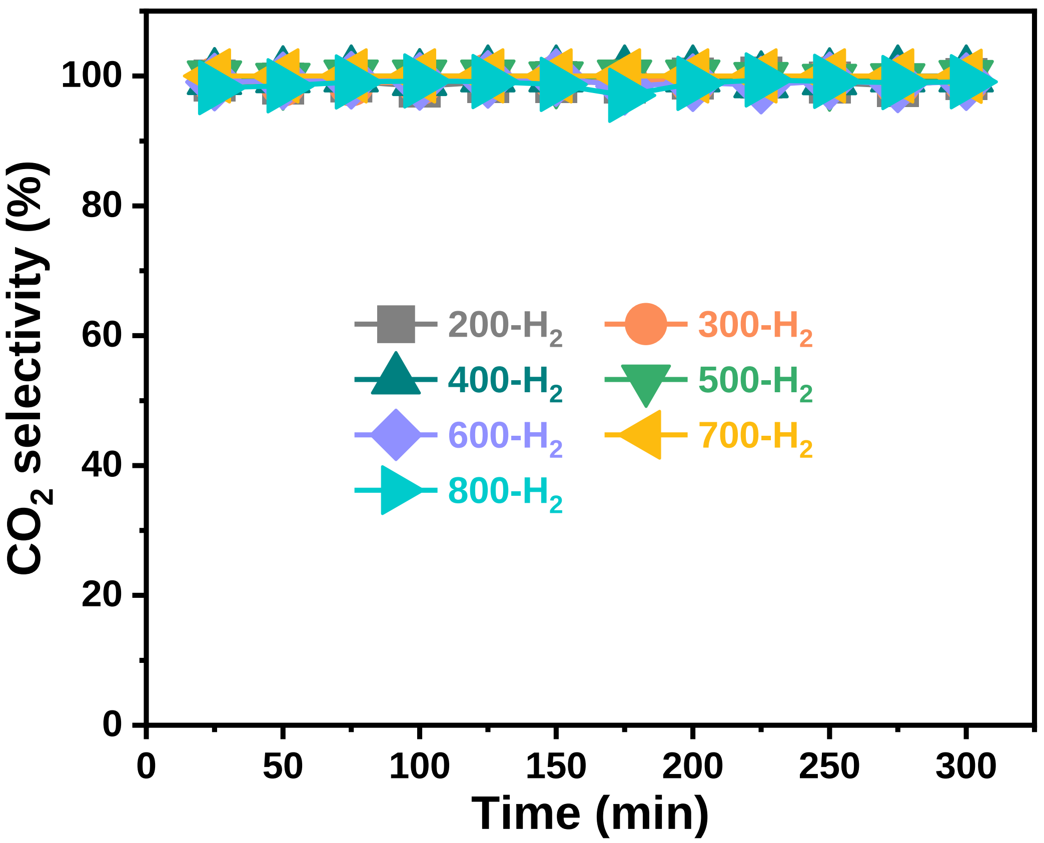

Figure S22. CO_2 selectivity as a function of time over Pt@NFM-SiO_2 at different reduction temperatures.

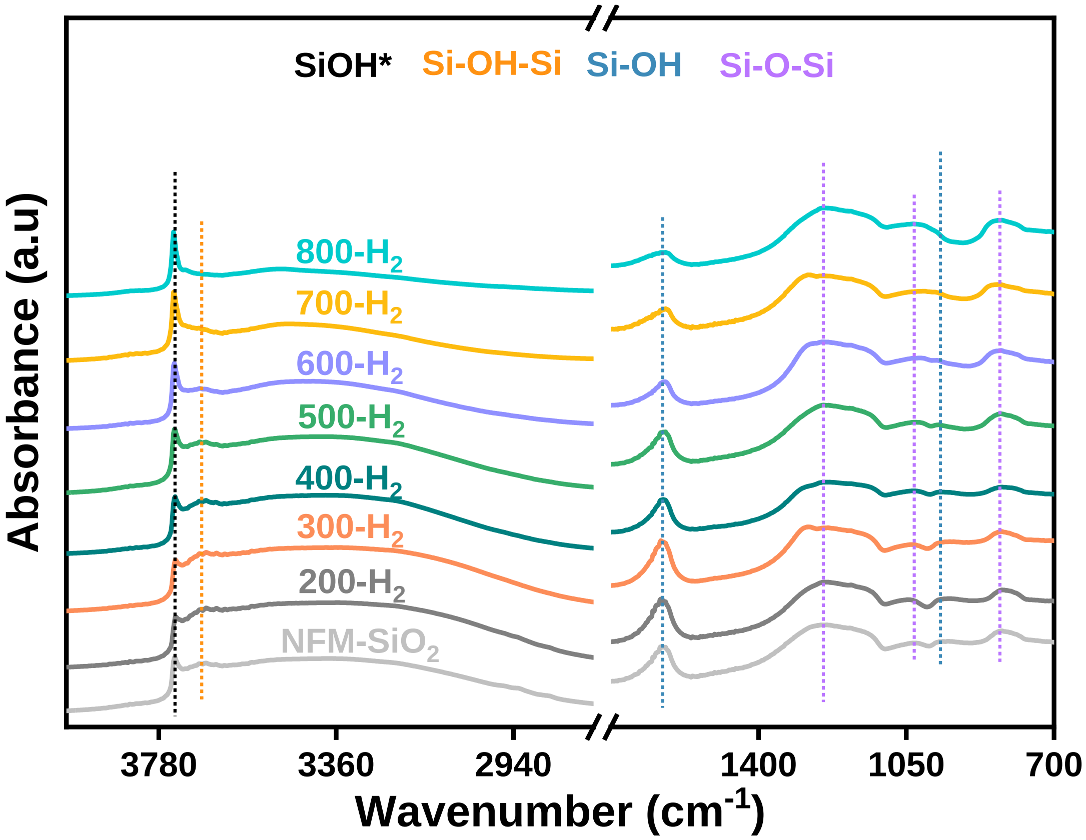

Figure S23. FT-IR spectra of Pt@NFM-SiO_2 at different reduction temperatures.

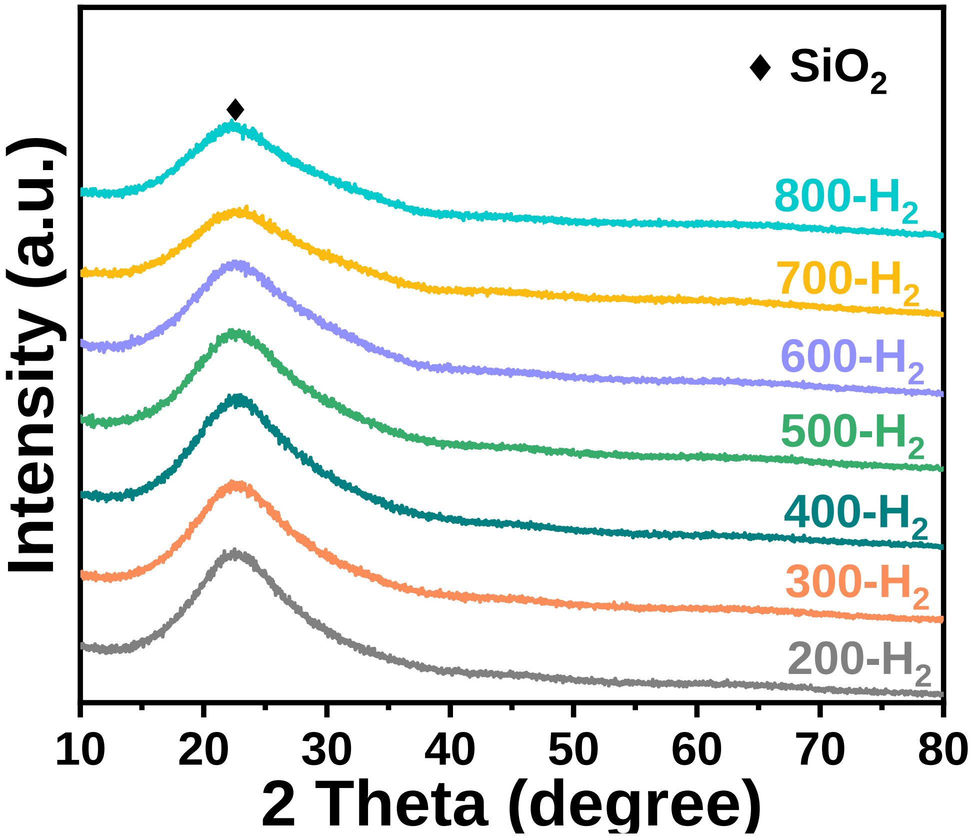

Figure S24. XRD of Pt@NFM-SiO_2 under different reduction temperatures.

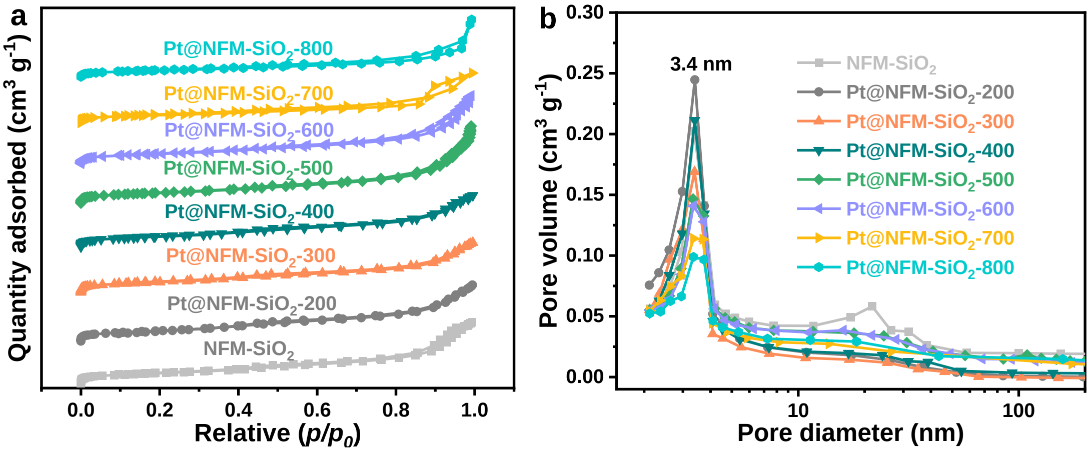

Figure S25. a) N_2 adsorption/desorption isotherms and b) pore size distribution curve of Pt@NFM-SiO_2-X (X denotes reduction temperature) and NFM-SiO_2.

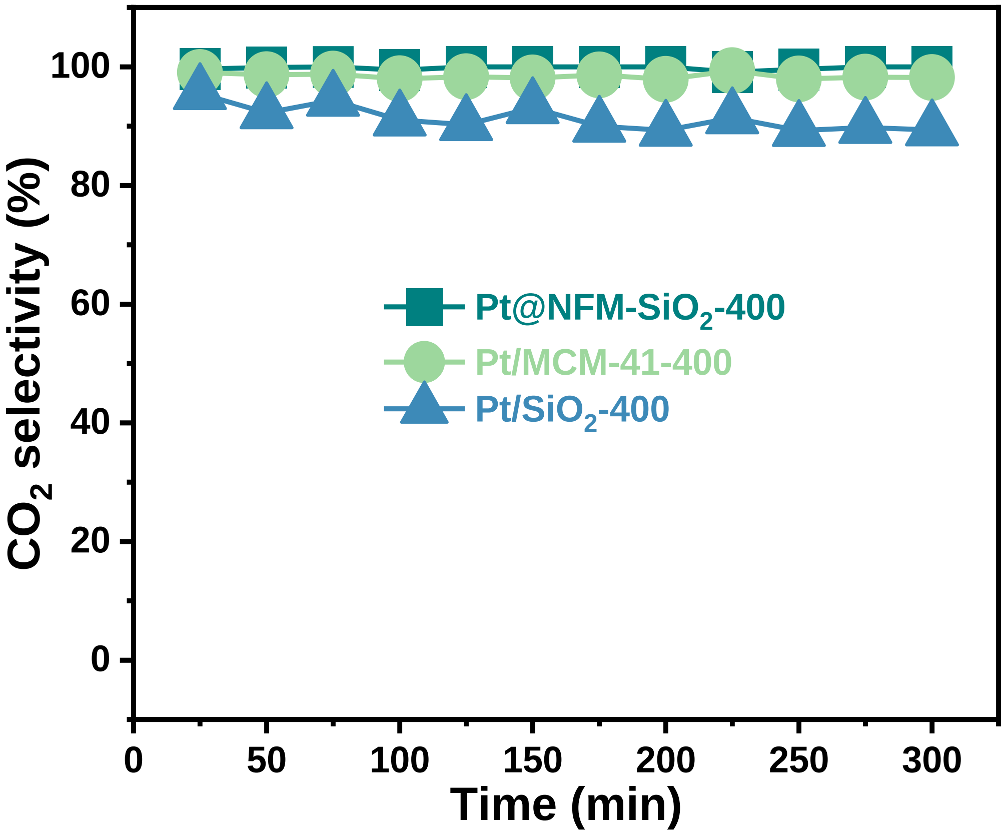

Figure S26. CO_2 selectivity as a function of time over Pt@NFM-SiO_2-400, Pt/MCM-41-400, and Pt/SiO_2-400.

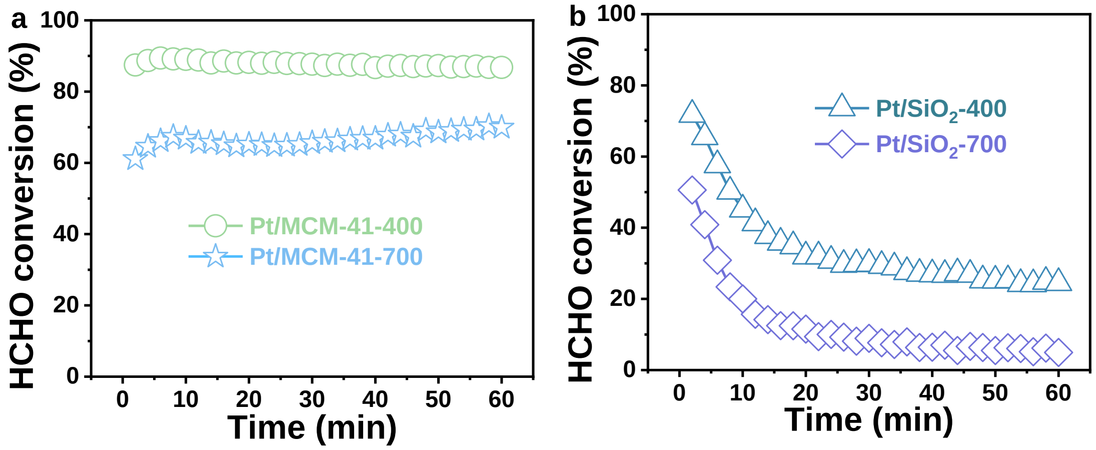

Figure S27. HCHO oxidation over Pt/MCM-41-400, Pt/MCM-41-700, Pt/SiO_2-400, and Pt/SiO_2-700.

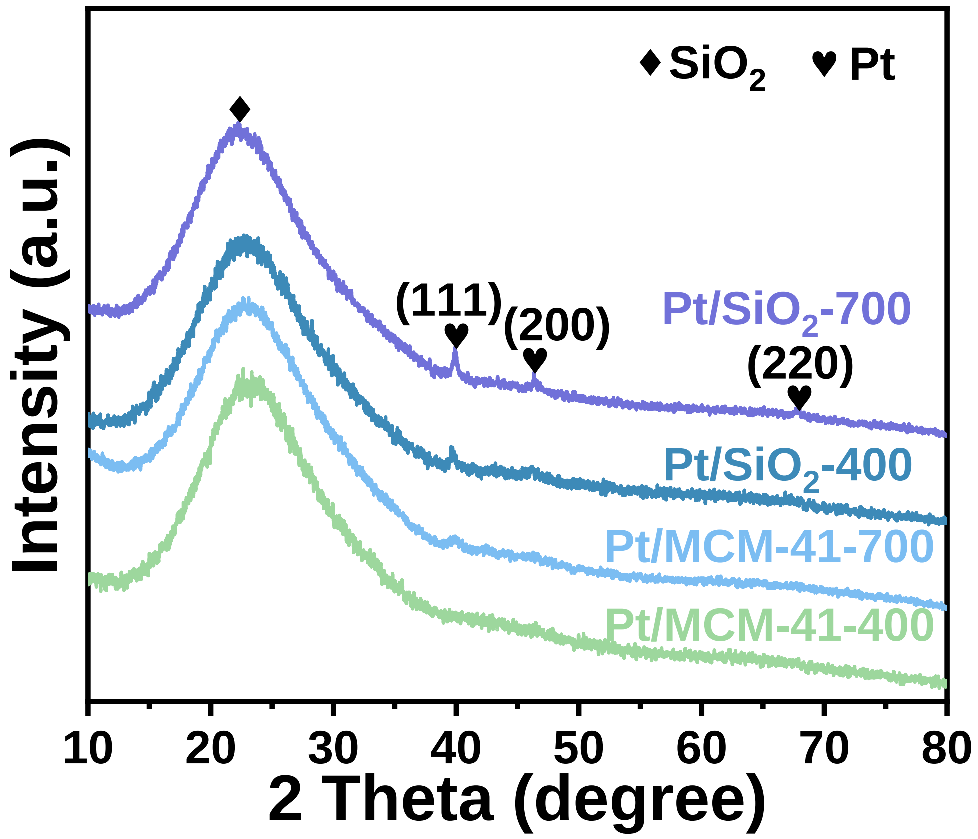

Figure S28. XRD of Pt/MCM-41-400, Pt/MCM-41-700, Pt/SiO_2-400, and Pt/SiO_2-700.

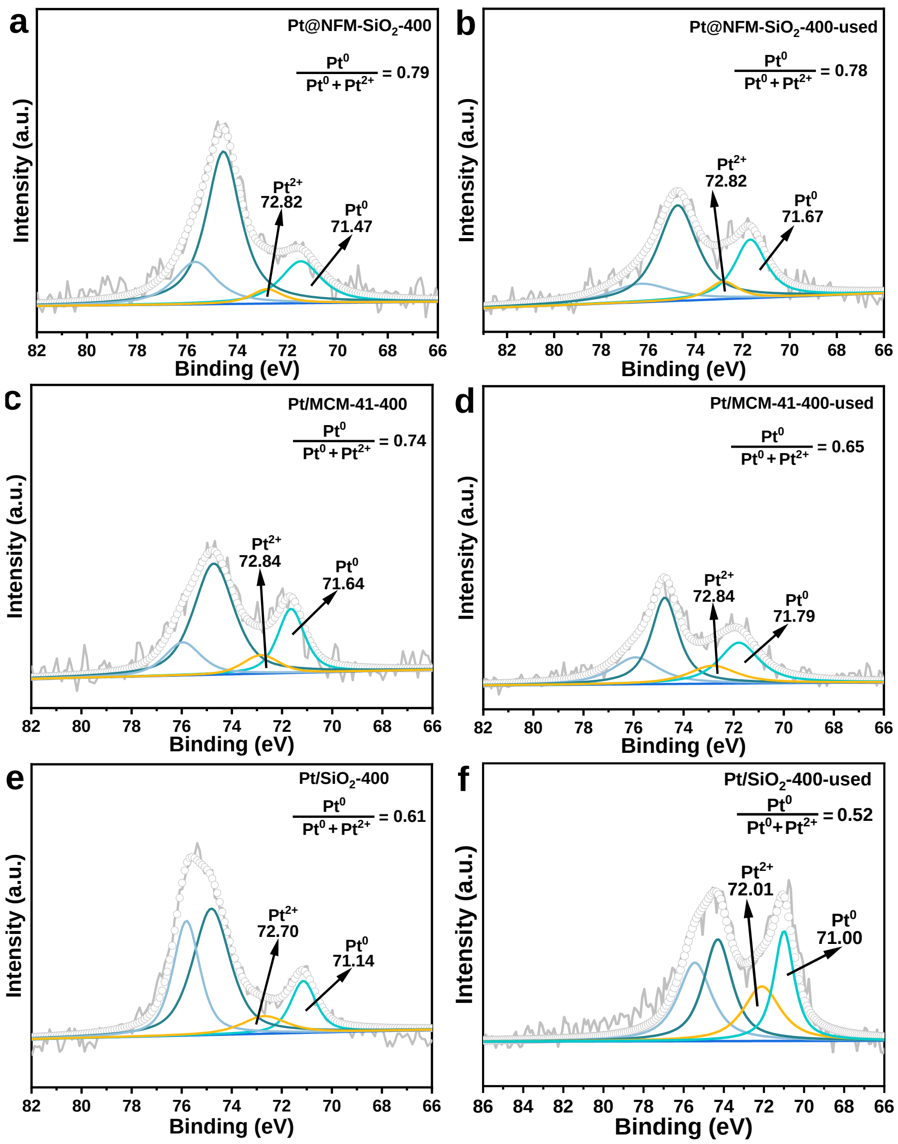

Figure S29. XPS spectra of Pt 4f over a) Pt@NFM-SiO_2-400, b) Pt@NFM-SiO_2-400-used, c) Pt/MCM-41-400, d) Pt/MCM-41-400-used, e) Pt/SiO_2-400, and f) Pt/SiO_2-400-used.

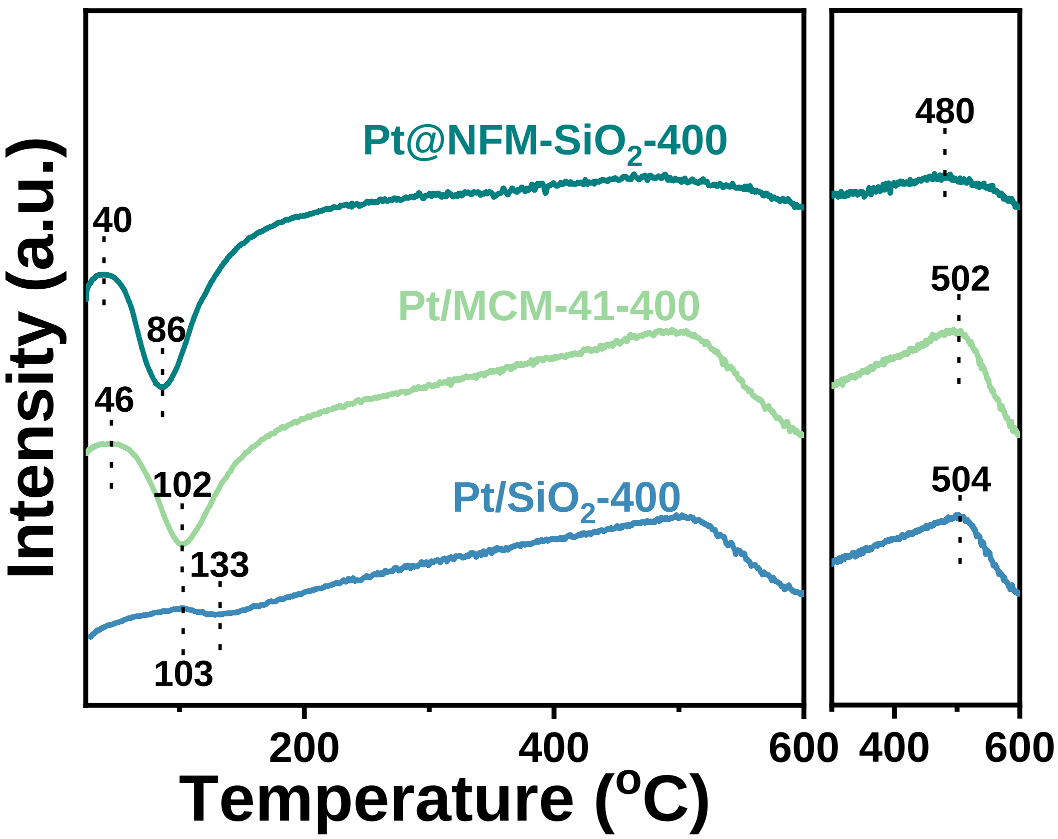

Figure S30. H_2-TPR of Pt@NFM-SiO_2-400, Pt/MCM-41-400, and Pt/SiO_2-400.

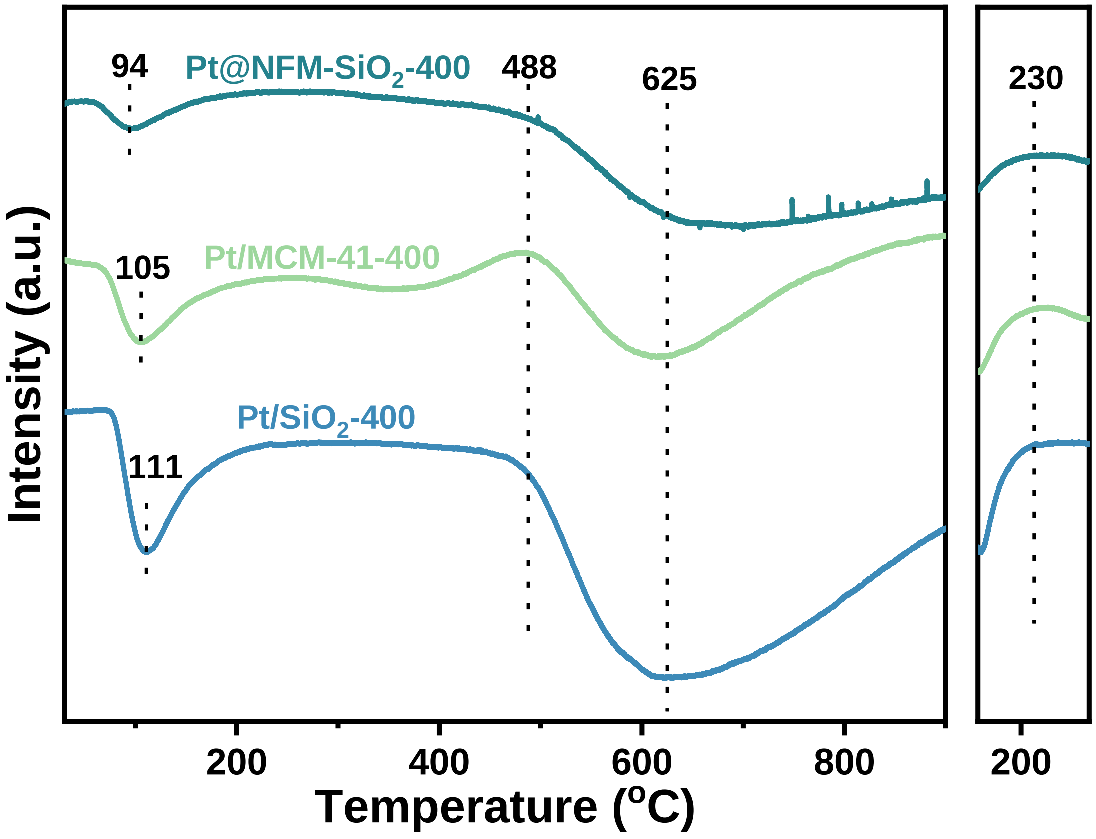

Figure S31. O_2-TPO of Pt@NFM-SiO_2-400, Pt/MCM-41-400, and Pt/SiO_2-400.

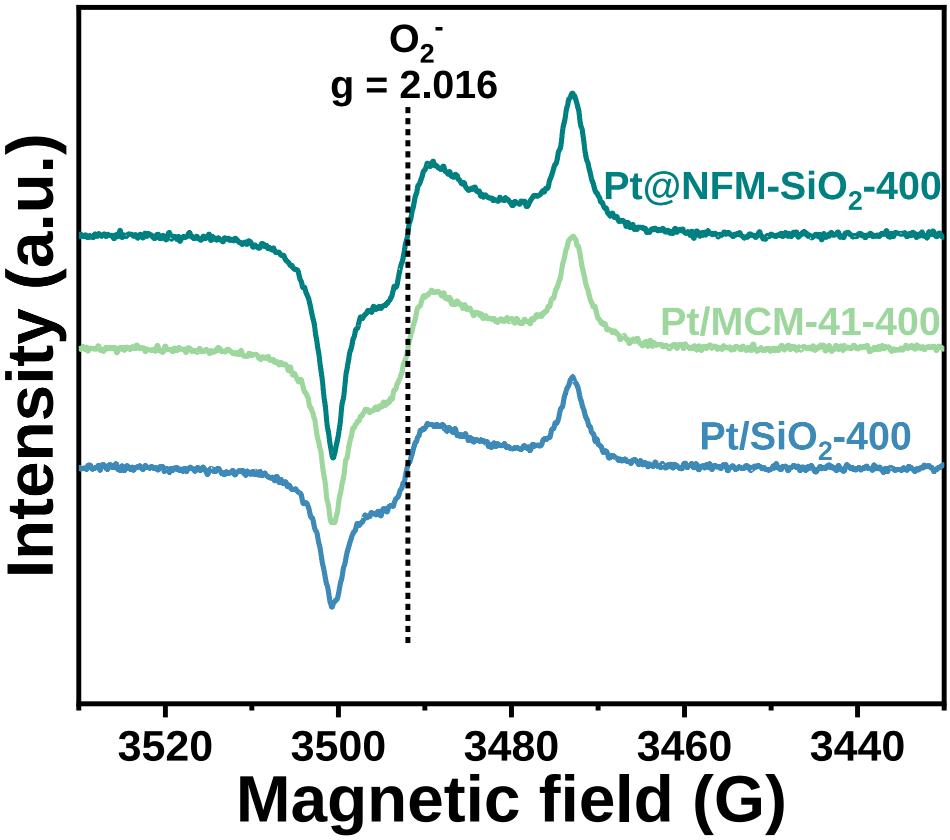

Figure S32. EPR spectra of Pt@NFM-SiO_2-400, Pt/MCM-41-400, and Pt/SiO_2-400.

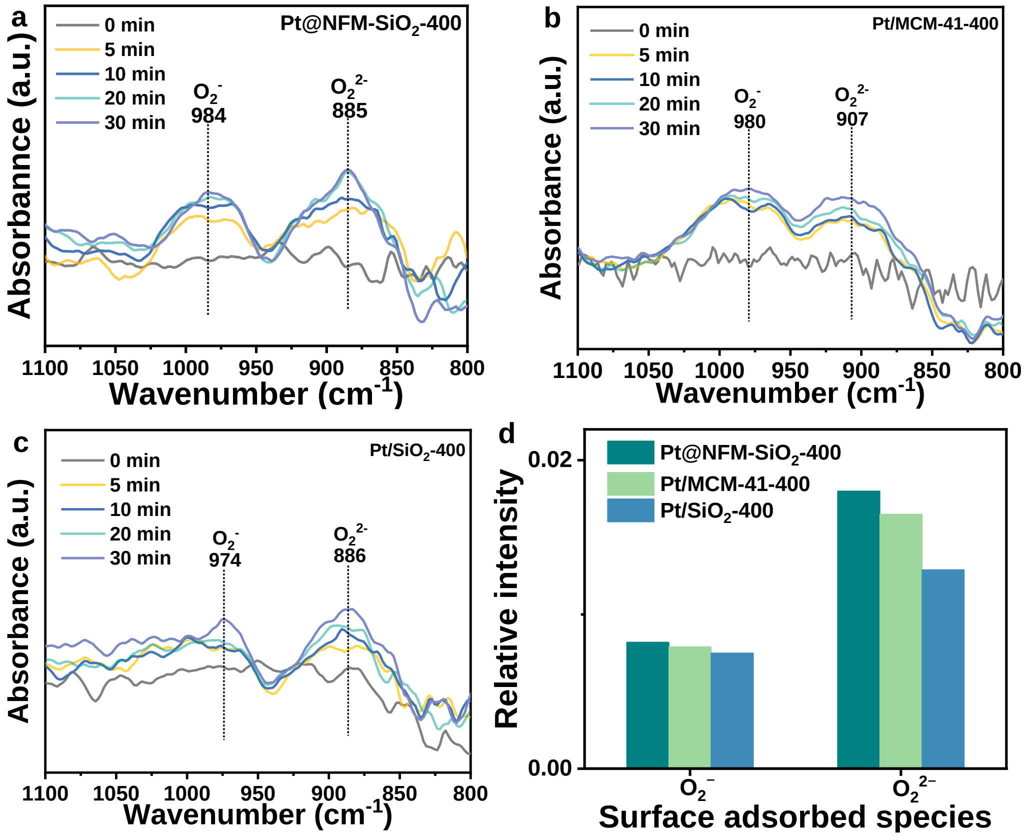

Figure S33. O_2-DRIFTS spectra of a) Pt@NFM-SiO_2-400, b) Pt/MCM-41-400, and c) Pd/SiO_2-400 exposed in a 5% O_2 + Ar flow at 30 ºC. d) Related dynamic evolution of the integrated area of surface reactive oxygen species at 30 min.

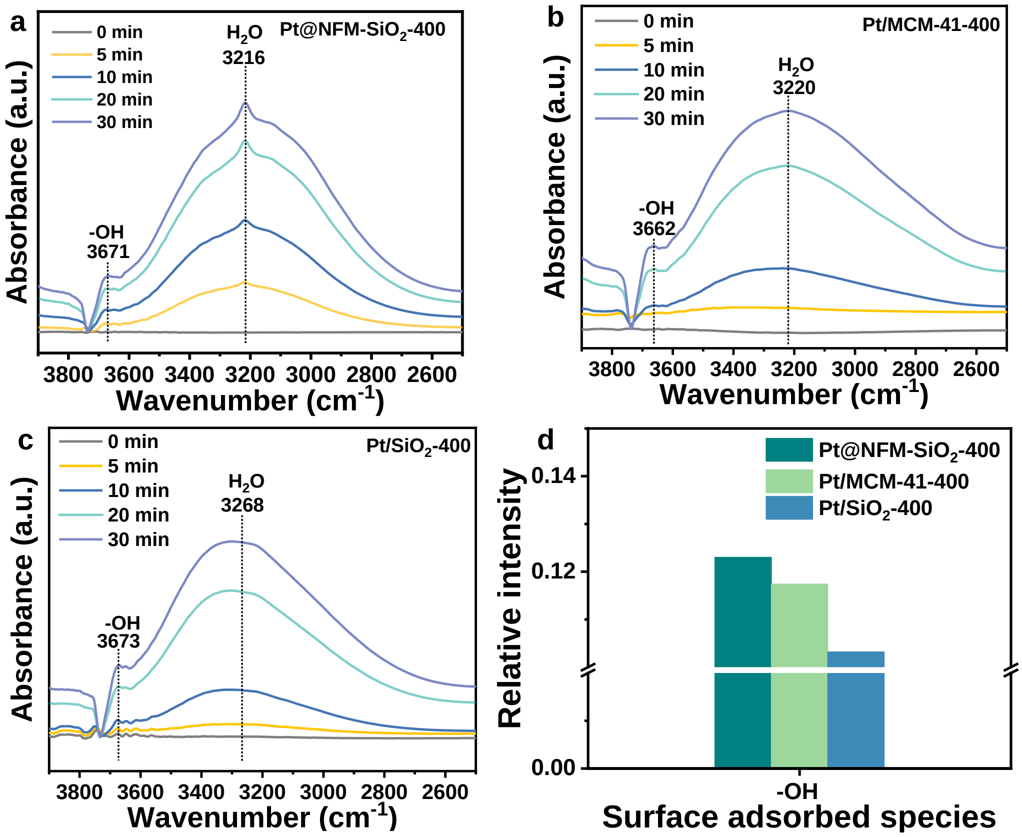

Figure S34. H_2O-DRIFTS spectra of a) Pt@NFM-SiO_2-400, b) Pt/MCM-41-400, and c) Pd/SiO_2-400 exposed in a 5% O_2 + Ar flow at 30 °C. d) Related dynamic evolution of the integrated area of surface –OH species.

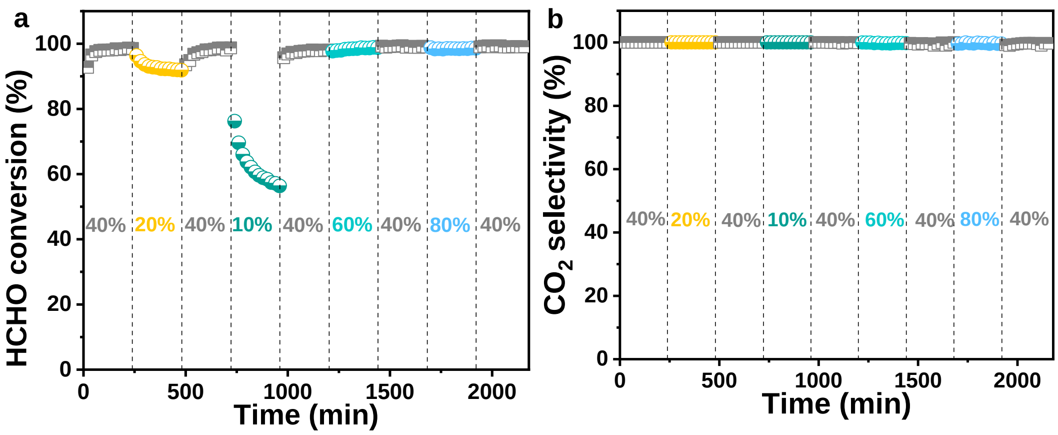

Figure S35. a) HCHO conversion and b) CO_2 selectivity as a function of time over Pt@NFM-SiO_2-400 under different humidity.

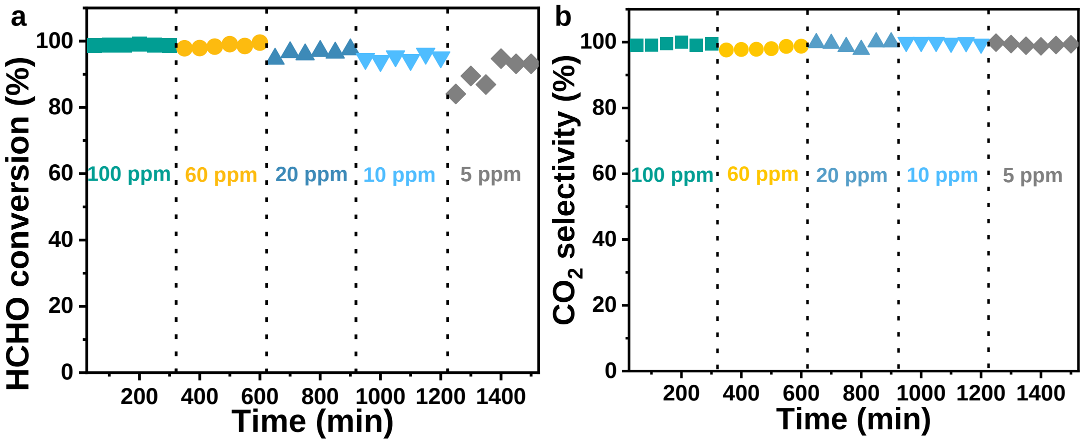

Figure S36. a) HCHO conversion and b) CO_2 selectivity as a function of time over Pt@NFM-SiO_2-400 at different concentration.

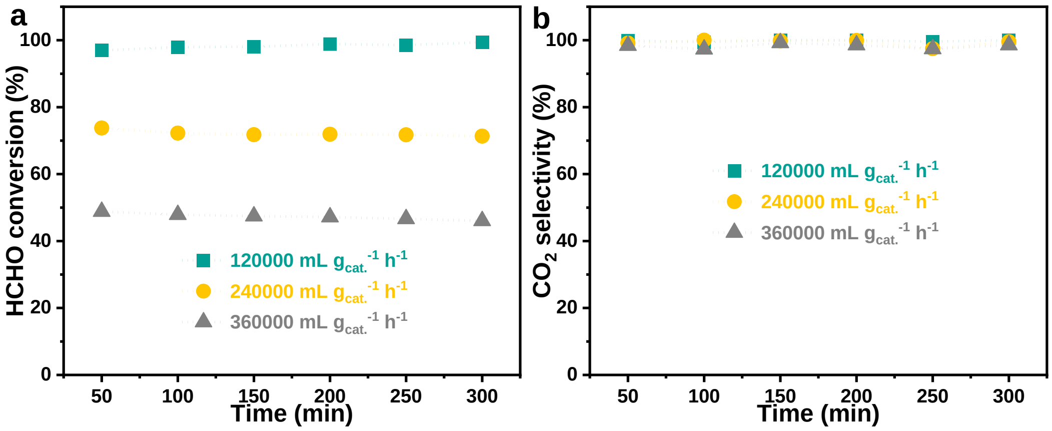

Figure S37. a) HCHO conversion and b) CO_2 selectivity as a function of time over Pt@NFM-SiO_2-400 under different WHSV.

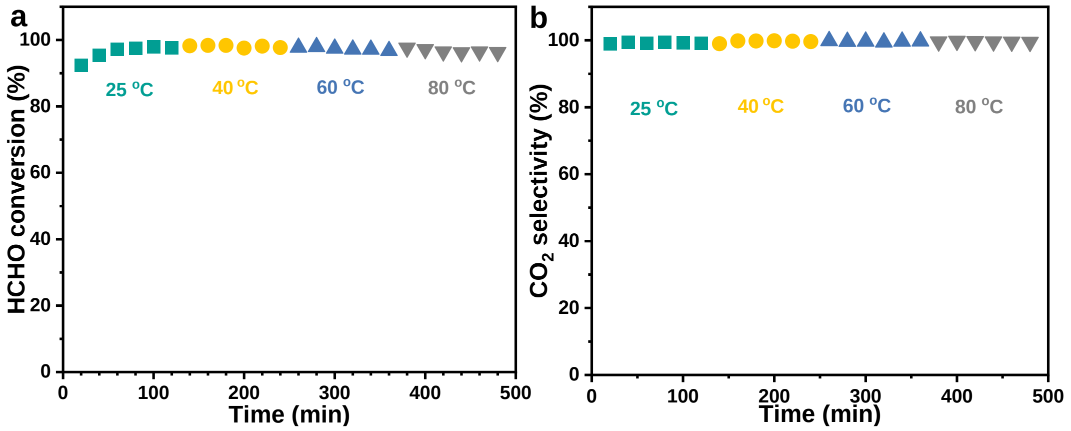

Figure S38. a) HCHO conversion and b) CO_2 selectivity as a function of time over Pt@NFM-SiO_2-400 under different temperature.

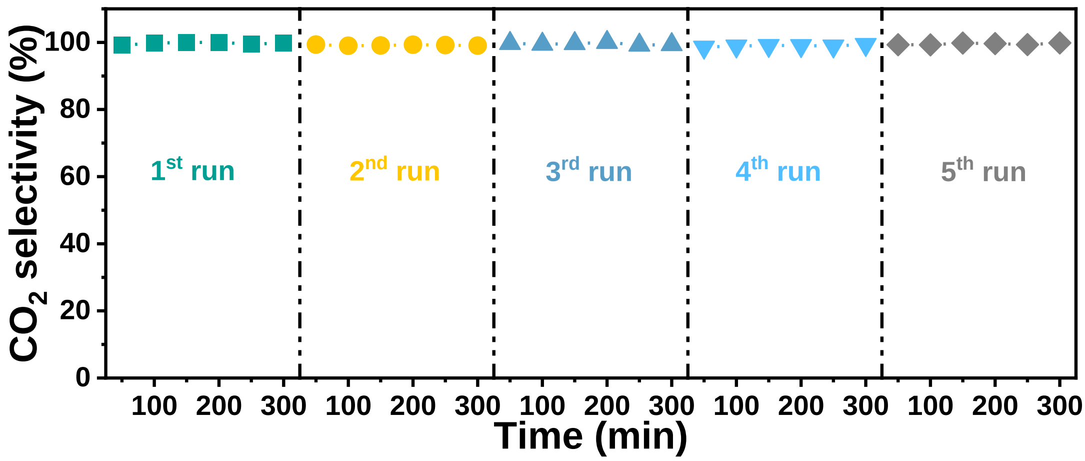

Figure S39. CO_2 selectivity of HCHO oxidation over Pt@NFM-SiO_2-400 for cycling test.

References

[1] Z. Xia, H. Zhang, K. Shen, Y. Qu, Z. Jiang, *Phys. B Condens. Matter* **2018**, *542*, 12-19.

[2] H. Funke, M. Chukalina, A.C. Scheinost, *J. Synchrotron Radiat.* **2007**, *14*, 426-432.

[3] Ravel, B. & Newville, M. *J. Synchrotron Radiat.* **2005**, *12*, 537-541.

[4] Z. Hu, Z. Wang, Y. Guo, L. Wang, Y. Guo, J. Zhang, W. Zhan, *Environ. Sci. Technol.* **2018**, *52*, 9531-9541.

[5] G. Kresse, J. Furthmüller, *Comput. Mater. Sci.* **1996**, *6*, 15-50.

[6] G. Kresse, J. Furthmuller, *Phys. Rev. B* **1996**, *54*, 11169-11186.

[7] J.P. Perdew, K. Burke, M. Ernzerhof, *Phys. Rev. Lett.* **1996**, *77*, 3865-3868.

[8] P. E. Blochl, *Phys. Rev. B* **1994**, *50*, 17953.

[9] W. Reckien, F. Janetzko, M.F. Peintinger, T. Bredow, *J. Comput. Chem.* **2012**, *33*, 2023-2031.

[10] J. Ye, M. Zhou, Y. Le, B. Cheng, J. Yu, *Appl. Catal. B Environ. Energy* **2020**, *267*, 118689.

[11] T. Dong, J. Ji, L. Yu, P. Huang, Y. Li, Z. Suo, B. Liu, Z. Hu, H. Huang, *JACS Au* **2023**, *3*, 1230-1240.

[12] C. Wang, Y. Li, L. Zheng, C. Zhang, Y. Wang, W. Shan, F. Liu, H. He, *ACS Catal.* **2021**, *11*, 456-465.

[13] C. Wang, Y. Li, C. Zhang, X. Chen, C. Liu, W. Weng, W. Shan, H. He, *Appl. Catal. B Environ. Energy* **2021**, *282*, 119540.

[14] X. Ye, H. Wang, Y. Lin, X. Liu, L. Cao, J. Gu, J. Lu, *Nano Res.* **2019**, *12*, 1401-1409.

[15] A. I. Serykh, O. P. Tkachenko, V. Yu. Borovkov, V. B. Kazansky, M. Beneke, N. I. Jaeger, G. Schulz-Ekloff, *Phys. Chem. Chem. Phys.* **2000**, *2*, 5647-5652.

[16] Y. Li, X. Chen, C. Wang, C. Zhang, H. He, *ACS Catal.* 2018, **8**, 11377-11385.
